# Overexpression of a peach CBF gene in apple: a model for understanding the integration of growth, dormancy, and cold hardiness in woody plants

**DOI:** 10.3389/fpls.2015.00085

**Published:** 2015-02-27

**Authors:** Michael Wisniewski, John Norelli, Timothy Artlip

**Affiliations:** United States Department of Agriculture – Agricultural Research ServiceKearneysville, WV, USA

**Keywords:** freezing tolerance, fruit trees, *DAM* genes, *CBF* genes, bud break, *DELLA* genes, *EBB* genes, *Malus* × *domestica*

## Abstract

The timing of cold acclimation and deacclimation, dormancy, and budbreak play an integral role in the life cycle of woody plants. The molecular events that regulate these parameters have been the subject of much study, however, in most studies these events have been investigated independently of each other. Ectopic expression of a peach *CBF* (*PpCBF1)* in apple increases the level of both non-acclimated and acclimated freezing tolerance relative to the non-transformed control, and also inhibits growth, induces early bud set and leaf senescence, and delays bud break in the spring. The current study examined differences in the seasonal expression of genes (*CBF, DAM, RGL*, and *EBB*) that have been reported to be associated with freezing tolerance, dormancy, growth, and bud break, respectively, in the *PpCBF1* T166 transgenic apple line and the non-transformed M.26 control. Results indicated that expression of several of these key genes, including *MdDAM*, *MdRGL*, and *MdEBB* was altered in transgenic T166 trees relative to non-transformed M.26 trees. In particular, several putative *MdDAM* genes, associated with the dormancy-cycle in other species of woody plants in the Rosaceae, exhibited different patterns of expression in the T166 vs. M.26 trees. Additionally, for the first time a putative APETALA2/Ethylene-responsive transcription factor, originally described in poplar and shown to regulate the timing of bud break, was shown to be associated with the timing of bud break in apple. Since the overexpression of *PpCBF1* in apple results in a dramatic alteration in cold acclimation, dormancy, and growth, this transgenic line (T166) may represent a useful model for studying the integration of these seasonal life-cycle parameters.

## Introduction

The timing of cold acclimation and deacclimation, the onset and release from dormancy, as well as the timing of bud break play an integral role in the life cycle of woody plants and their adaptation to the external environment (Rios et al., 2014). These parameters are especially important for perennial fruit crops where the timing and regulation of these events play a critical role in the selection of cultivars that are appropriate for use in a specific growing region and where the inability of a cultivar to adequately respond to regional environmental conditions can result in the complete loss of a harvestable fruit crop or an entire planting. The molecular events that regulate these parameters have been the subject of much study and numerous reviews on these topics have been published (Rohde et al., 2007; Ruttink et al., 2007; Hänninen and Tanino, 2011; Cooke et al., 2012; Rios et al., 2014). In most studies, however, these events have been investigated independently of each other, with the focus being on either dormancy, cold acclimation, or growth regulation, and the systems used have been advantageous for providing information specific to one of these events. While this approach has provided a wealth of essential information, it is also important to note that these events do not occur in isolation but rather as an integrated part of the entire life cycle of the plant species being investigated. For instance, it has been well established that woody plants cannot cold acclimate to their full potential unless they are dormant and that it is difficult for plants to reacclimate to any substantial degree once the plant is released from endodormancy. The loss of freezing tolerance is also intimately associated with the onset of growth (bud break), a parameter that lies outside the realm of endodormancy and is regulated by the accumulation of heat units, a requirement which is determined by an interaction between the genetics of a species and the environment. Epigenetics, in addition to genetic makeup, has also been recognized to play a significant role in the regulation of these phenological events (Leida et al., 2012; Rios et al., 2014), further adding to the complexity.

Several specific transcription factors and regulatory genes have been demonstrated to play an important role in one or more of the processes mentioned above. *CBF* genes have been shown to play an integral role in the induction of freezing tolerance of both herbaceous and woody plants (Gilmour et al., 1998, 2000; Thomashow et al., 2001; Welling and Palva, 2006; Wisniewski et al., 2014). The regulation of freezing tolerance by *CBF* in woody plants, however, appears to be more complex than in herbaceous plants and the role of specific *CBF* genes can also vary (Benedict et al., 2006; Xiao et al., 2006; Welling and Palva, 2008; Wisniewski et al., 2014). Additionally, *CBF* expression can alter parameters other than freezing tolerance, such as growth and the timing and development of flowering (Achard et al., 2008). Collectively, these reports add to the complexity of the regulation of freezing tolerance by *CBF* genes and also suggest that integration, cross-talk, and some degree of overlap may exist in the regulation of key developmental aspects of plants.

A variety of genetic components that contribute to the intricate regulation of dormancy have been reported (Rohde and Bhalerao, 2007; Ruttink et al., 2007; Cooke et al., 2012). Among these regulatory components, *DAM* (*Dormancy Associated MADS-box*) genes have been reported to play an intimate role in controlling dormancy in fruit trees within the Rosaceae, and other plant species (Bielenberg et al., 2008; Li et al., 2009; Horvath et al., 2010; Falavigna et al., 2014). In particular, *DAM5* and *DAM6* have been highlighted as being associated with the onset and release of dormancy in peach (*Prunus persica* L. Batsch) (Yamane et al., 2011). A natural mutation of one of the *DAM* genes in peach has resulted in a non-dormant evergreen peach (Rodriguez et al., 1994; Bielenberg et al., 2008). Several *DAM* genes have also been associated with the dormancy in apricot, *Prunus mume* (Sasaki et al., 2011) Epigenetic regulation of *DAM* genes has been reported (Leida et al., 2012). The role of *DAM* genes in the regulation of dormancy, however, does not appear to be universal (Rios et al., 2014). For example, *DAM* genes do not map to the QTL associated with dormancy in Rohde et al. (2011). It is also important to note that genes directly regulating endormancy may not be the same genetic components regulating time to bud break, once plants have acquired sufficient chill units and become ecodormant. In this regard, Yordanov et al. (2014) have recently reported that the expression of an early bud-break1 (*EBB1)* gene in poplar plays a major role in regulating the timing of bud break.

*RGL* genes, which code for DELLA proteins, act to restrain growth, whereas GA promotes growth by overcoming DELLA-mediated growth restraint (Achard and Genschik, 2009; Claeys et al., 2014). *CBF* genes have been reported to influence the expression of *RGL* genes and this interrelationship has been used to explain the impact of *CBF* genes on growth (Achard et al., 2008). Thus, the interaction of *CBF* with *RGL* genes may play a role in the interaction between growth and deacclimation. While plausible, this still needs to be demonstrated and how one process (cold acclimation vs. growth) becomes dominant still remains to be explored.

Wisniewski et al. (2011) reported that the ectopic expression of a peach *CBF* (*PpCBF1)* in apple not only increased the level of both non-acclimated and acclimated freezing tolerance in the transgenic apple (T166), relative to the non-transformed control (M.26), but also inhibited growth, and surprisingly rendered the T166 plants sensitive to short day (SD) photoperiod, and induced early leaf senescence and bud set, again relative to the non-transformed M.26 plants. The observed sensitivity to SD was novel and unexpected since apples are typically not sensitive SD in terms of inducing growth cessation (Heide and Prestrud, 2005). Three years of field studies with the T166 plants further confirmed that, relative to the control, the transgenic apple line had increased level of freezing tolerance, reduced growth (current year and main stem diameter growth), set bud earlier, experienced earlier leaf senescence, and later bud break in the spring (Artlip et al., 2014). Thus, this transgenic line may serve as a model for studying the integration of the regulation of freezing tolerance, dormancy, bud break, and growth in woody plants. The current study examined differences in the seasonal expression of genes (*CBF, DAM, RGL*, and *EBB*) that have been reported to be associated with freezing tolerance, dormancy, bud break, and growth in the T166 line of transgenic apple and its non-transformed M.26 control. The purpose of the present study was to characterize the expression of all five different apple *CBF* genes in response to a cold acclimating conditions and to determine if the overexpression of *CBF* also modified the expression of genes that have been reported in the literature to modify dormancy, bud break, and growth.

## Materials and methods

### Plant material

“Malling 26” (M.26) is a standard dwarfing apple rootstock. T166 (PpCBF1) transgenic line was initially described by Wisniewski et al. (2011). Briefly, M.26 leaves underwent *Agrobacterium*-mediated transformation with a vector consisting of a pBINPLUSARS (Belknap et al., 2008) backbone and the peach (*Prunus persica*) *PpCBF1* gene driven by a dual 35 s enhancer segment derived from pRTL2 (Restrepo et al., 1990). Plants were maintained in tissue culture, roots initiated, plantlets grown successively in growth chambers and greenhouse, before being planted in October, 2010 at the Appalachian Fruit Research Station, USDA-ARS, Kearneysville, WV per Artlip et al. (2014). A commercial scion cultivar, “CrimsonCrisp,” was used in a limited fashion to compare expression of *RGL* genes in a scion variety vs. the non-transformed (M.26) and transformed (T166) rootstock cultivar. The trees were also located on the grounds of the research station and were planted 07 May, 2007 on Bud-9 rootstock and subjected to conventional management practices.

### Growth and phenology of field-grown M.26 and T166 trees

Growth measurements were taken monthly during the growing season (March to November) during 2013. Caliper (stem diameter) data were taken at a point 30 cm above the ground on the main stem, and current season's shoot lengths were taken from the terminal bud scar of the previous year's growth to the tip of the main stem; cumulative data are presented. Dates of bud break for each tree were recorded during spring 2013. Percent bud break was determined as follows: three shoots on each of three trees of M.26 and T166 were tagged and bud break from 20 individual lateral buds from the terminal bud were tracked. The range of dates of leaf loss were recorded in autumn, 2012. Two-sample independent *t*-tests were used to determine significance between the calculated means. For the current year shoot data, *n* = 10 for the transgenic T166 line and *n* = 7 for the non-transformed M.26 trees. For the stem caliper data, *n* = 13 for the transgenic T166 line and *n* = 7 for the non-transformed M.26 trees. Differences in sample number between the lines were due to different numbers of trees in the original planting and the loss of some trees, mainly M.26, over the last 4 years.

### Tissue collection

Small branches were removed from the trees for bark or axillary bud collection. Bark tissue (phloem, cambium and epidermis) was destructively sampled from M.26 and T166 trees on a monthly basis in 2013. Axillary buds were collected bi-weekly from M.26 and T166 trees from January through April, 2013. The tissues were flash-frozen in liquid N_2_, lyophilized, and stored at −20°C until use.

### M.26 cold acclimation experiment

One-year-old M.26 trees were propagated in tissue culture, rooted, and grown in a glass house as per Wisniewski et al. (2011). Ten tress were transferred to a PGV36 growth chamber (Conviron, Winnipeg, MN, Canada) at 4°C, 200 μmole photons M^−2^ s^−1^, 8 h light/16 h dark for cold treatment and acclimation. Leaves were removed from three trees each at 0, 15, 30 min, 1, 2, 4, 8, 12, 24, 48, 96 h, 1 and 3 weeks. The leaves were immediately flash frozen in liquid N_2_ and stored at −80°C until use.

### RT-qPCR

Total RNA was isolated from leaf and bark tissues using Concert Plant RNA Reagent (Invitrogen, Carlsbad, CA, USA), treated with DNase (Turbo DNA-free Kit; Ambion, Austin, TX, USA) and then were diluted based on preliminary testing for optimal response. Reverse transcriptase, quantitative polymerase chain reaction (RT-qPCR) analysis was performed using appropriate quantities of total RNA (per preliminary testing) as a template with the Power SYBR Green RNA-to-Ct 1-Step Kit (Applied Biosystems, Foster City, CA, USA) and 2.0 pmol of each primer per reaction; no-RT control reactions were included to test for residual DNA contamination. A ViiA 7 Real-Time PCR System (Applied Biosystems) was set to cycle as follows: cDNA synthesis at 48.0°C for 30 min; 95.0°C denaturation for 10 min; 40 cycles of 95.0°C for 15 s followed by 52.0–57.0°C (depending on primers used; Table S1) for 1 min; followed by ABI-specified hold and melt curve stages. Primers were verified for specificity by using genomic DNA template and assessing the resulting amplicon by agarose gel electrophoresis and qPCR with genomic DNA on the ViiA 7 Real-Time PCR System; all primers had a single band and single peak. Primer efficiency was also verified for all primer sets by qPCR analysis of a standard curve, constructed by serially diluting RNAs from the sample set starting at some concentration above what was used in unknown samples and ending at a concentration well below it. Three technical replicates were used for each biological replicate (tree, *N* = 3). The standard curve method was used to calculate transcript abundance relative to *EF1-α* as a reference gene (user bulletin no. 2; Applied Biosystems http://www3.appliedbiosystems.com/cms/groups/mcb_support/documents/generaldocuments/cms_040980.pdf). Other endogenous reference genes were also examined, but *EF1-α* was determined to be the best overall reference gene using NormFinder (Anderson et al., 2004). Normalized data were then re-normalized to the respective values at time 0, and the means taken from the biological replicates. Standard errors (SEs) were derived by dividing the standard deviations by the square root of *n*, where *n* = 9 (3 biological replicates × 3 technical replicates).

### Bioinformatic analyses

In order to identify putative gene families, apple *CBF* (Wisniewski et al., 2014), peach (Li et al., 2009), and pear (Saito et al., 2013) *DAM*, and poplar (Yordanov et al., 2014) *EBB* genes were used as queries in BLASTn (Thompson et al., 1994) analyses of the *Malus* × *domestica* genome v 1.0 at the Genome Database for Rosaceae (GDR; http://www.rosaceae.org). Sequences for apple *DELLA(RGL)* were based on the report by Foster et al. (2007). In order to identify cis-regulatory elements within putative promoter regions, *in silico* analysis of the 5′-UTRs (up to 1000 bp upstream of the putative translational start site) was conducted using PAN (http://plantpan.mbc.nctu.edu.tw/gene_group/index.php) (Chang et al., 2008), PLACE (http://www.dna.affrc.go.jp/PLACE/) (Higo et al., 1999), and PLANTCARE (http://bioinformatics.psb.ugent.be/webtools/plantcare/html/) (Lescot et al., 2002).

## Results

### Phenology and growth

**Figure 1:**
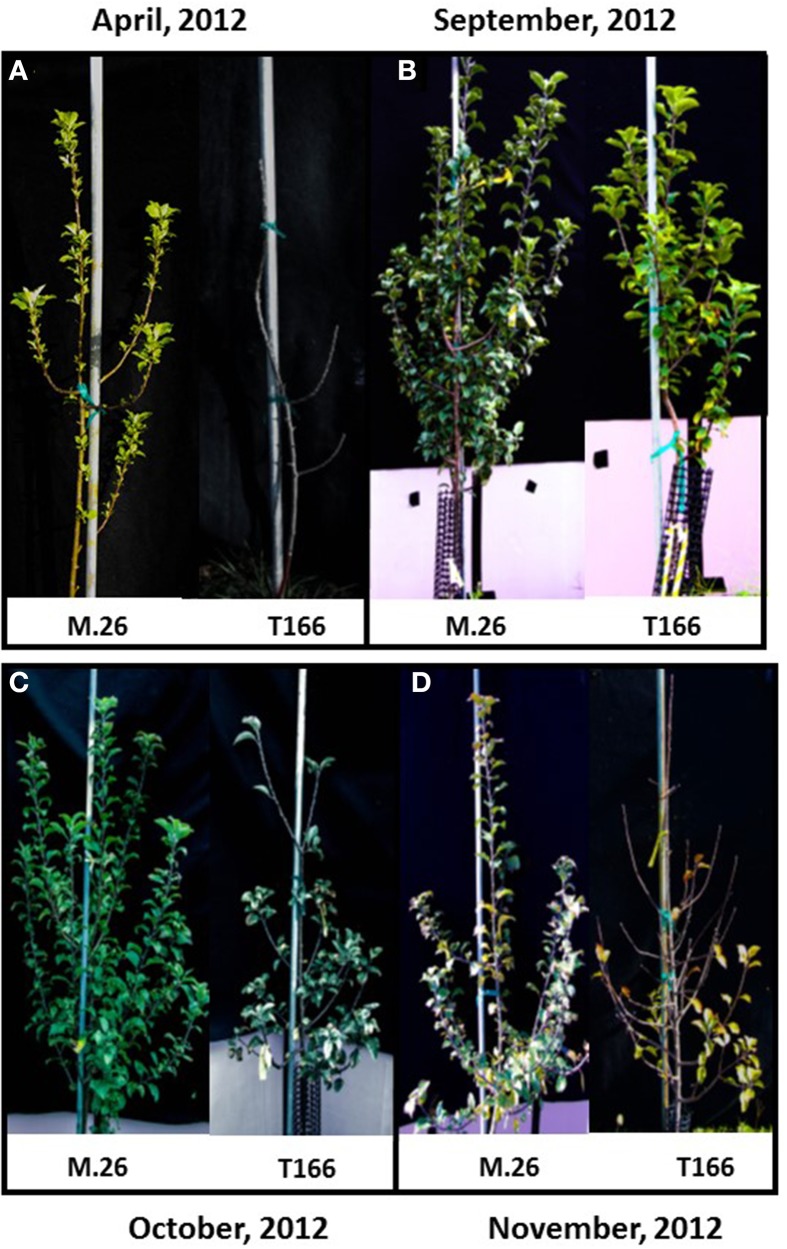
**M26 trees break bud earlier and enter dormancy later than T166 trees**. **(A)** Bud break. Photographs were taken in spring, 2012. **(B–D)** Leaf senescence and dormancy. Photographs were taken in September, October, and November and illustrate that Line T166 trees enter dormancy sooner as evidenced by leaf senescence and leaf drop occurring sooner than non-transformed M.26 trees.

**Figure 2:**
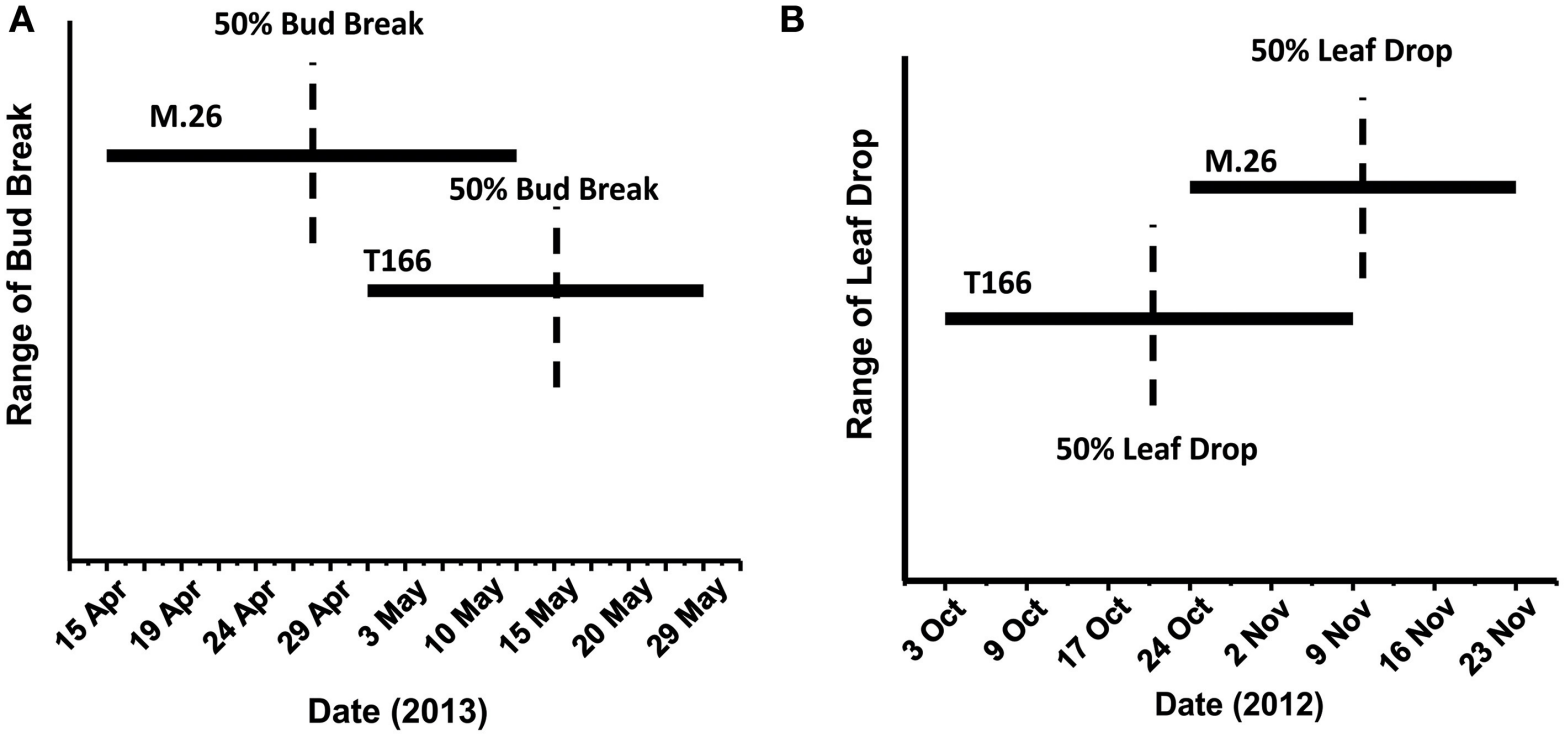
**Phenological disparities between non-transformed M.26 and Line T166**. **(A)** Bud break. Bud break was quantitatively assessed as the percent of 20 lateral buds from the terminal bud on three shoots and qualitatively assessed as the dates on which the terminal buds on these shoots broke dormancy. **(B)** Leaf drop. A quantitative assessment of percent leaf loss was made by estimating leaf loss over the entire tree compared to early September, when no leaf loss was evident, qualitatively assessed by date range when leaf loss was observed.

**Figure 3:**
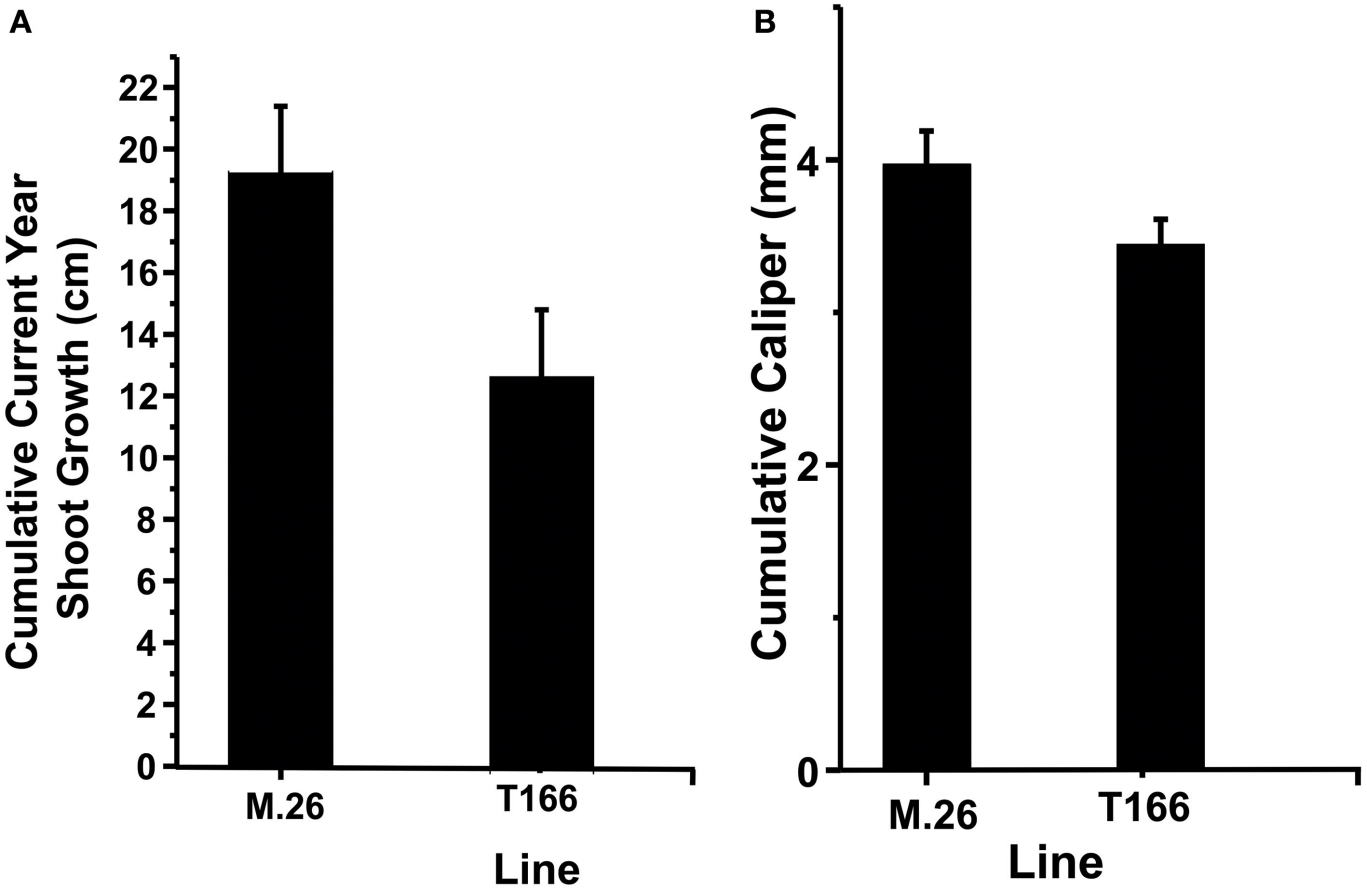
**T166 trees display reduced growth compared to M.26**. **(A)** Cumulative current year shoot growth. Growth was measured on the central axis from the previous season bud scar to the current terminal bud. The cumulative current year shoot growth was significantly different between the genotypes at the 0.05 level as assessed by a two-sample independent *t*-test. **(B)** Cumulative caliper (stem diameter) growth as measured 30 cm from the ground. The cumulative caliper growth was significantly different between the genotypes at the 0.05 level as assessed by a two-sample independent *t*-test.

**Figure 4:**
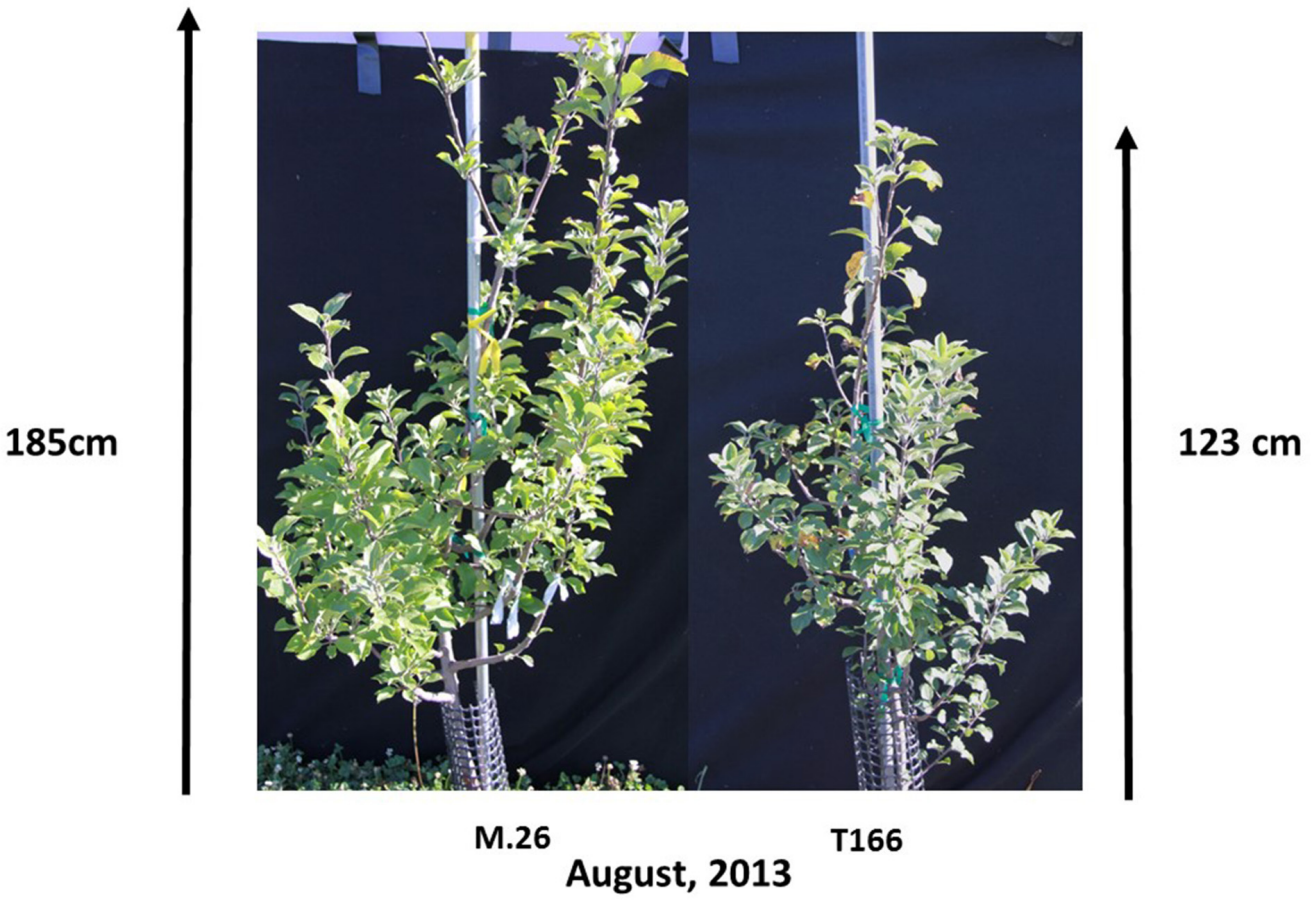
**Representative photographs of growth disparities between non-transformed M.26 and Line T166**. Non-transformed M.26 trees were 190 ± 10.2 cm overall height (*n* = 7 trees) while Line T166 trees were 130 ± 3.2 cm overall height (*n* = 12 trees).

As documented in a recent study (Artlip et al., 2014), field-planted, transgenic apple trees (T166) overexpressing a peach (*Prunus persica* L. Batsch.) *CBF* gene continued to exhibit delayed bud break and early senescence relative to the non-transformed, parent clone M.26 (Figure 1). The difference in the time of bud break and the onset of leaf senescence was very prominent between the two lines, being offset by approximately 2 weeks (Figure 2). Both current-year shoot growth (extension growth) and stem diameter (caliper growth) were reduced in T166 trees (Figure 3), as previously documented (Wisniewski et al., 2011; Artlip et al., 2014). Additionally, T166 trees typically had fewer lateral branches. The impact of the differences in growth between M.26 and T166 trees accumulated over several years resulting in T166 trees that were much smaller than the non-transformed M.26 trees (Figure 4). Average height for the T166 and M. 26 trees was 130 and 190 cm, respectively. This observation is significant since M.26 is known to be a dwarfing rootstock.

### Expression of apple CBF genes in response to cold acclimation

**Figure 5:**
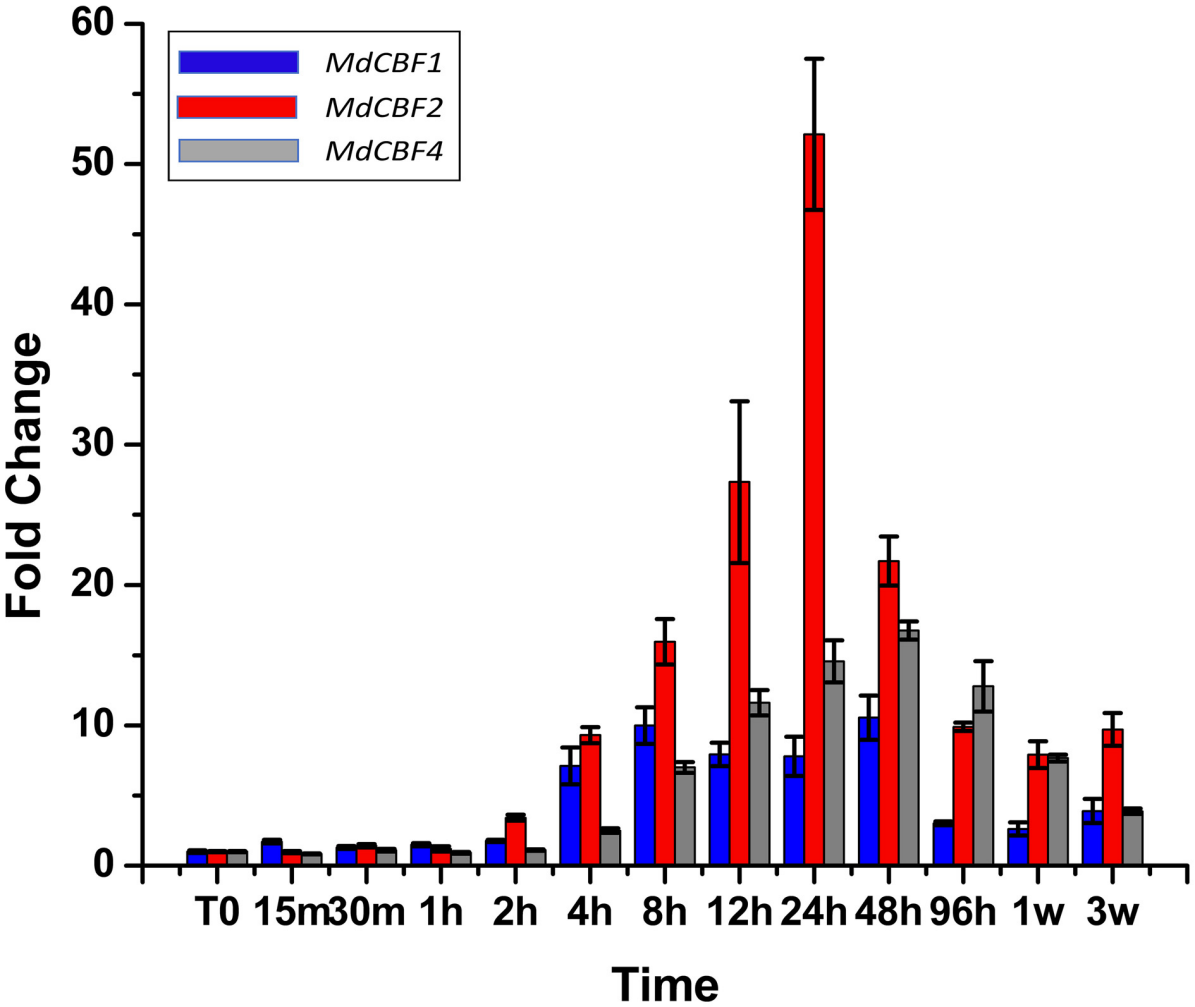
***MdCBF* transcript accumulation kinetics during cold-shock and cold-acclimation vary between genes**. Non-transformed M.26 trees were shifted to 4°C, 8 h light/16 h dark conditions, and leaves harvested at short time intervals (hours to days) or longer time intervals (weeks). *MdCBF1*, *2*, and *4* are shown; *MdCBF3* and *5* could not be detected by the primer sets used.

To date, the presence of five *CBF* genes (*MdCBFs1-5*) have been documented in the apple genome (Wisniewski et al., 2014; Figure S1). Previous research found that transcript abundance of two apple *CBF* genes (*MdCBF1* and *MdCBF2)* normally induced in response to low temperature are unaffected by ectopic expression of *PpCBF1* (Wisniewski et al., 2011). In the present analysis, an attempt was made to characterize the response of all five apple *CBF* genes to low temperatures over a short (96 h) and an extended (3 week) period of time (Figure 5) in non-transformed trees. Despite the use of numerous different primers (Table S1) and protocol adjustments, expression of *MdCBF3* and *MdCBF5* in response to low temperature (4°C, short day photoperiod) could not be documented. Only *MdCBF1*, *MdCBF2*, and *MdCBF4* appeared to be responsive to low temperature with *MdCBF2* exhibiting by far the strongest response as measured by fold change (Figure 5). A measurable induction was observed for *MdCBF1* and *MdCBF2* within 2 h after exposure of the M.26 trees to low temperature and peaked at 24 h. The pattern of induction of *MdCBF4* was slightly offset from the expression of the other two *CBF* genes. *MdCBF4* was induced after 4 h and appeared to peak at 48 h. After 24 h (*MdCBF1* and *MdCBF2)* or 48 h (*MdCBF4*) expression levels of all three induced *MdCBF* genes began to decrease, however, a measurable level of induction, relative to time 0, was still observable after 3 weeks.

### Dormancy and bud break candidate gene expression

**Figure 6:**
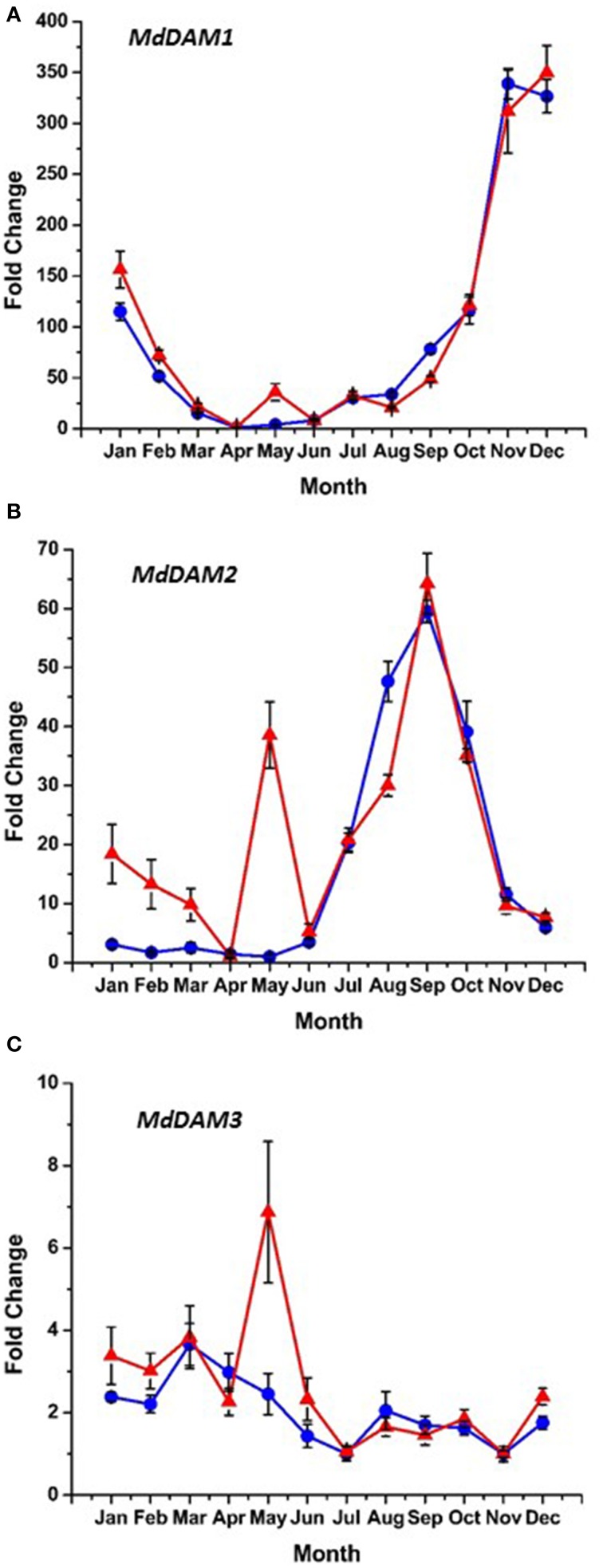
**Seasonal *MdDAM* transcript accumulation kinetics from bark tissue vary between genes**. **(A)** *MdDAM1*. **(B)** *MdDAM2*. **(C)** *MdDAM3*. Blue circles: non-transformed M.26; Red triangles: Line T166.

**Figure 7:**
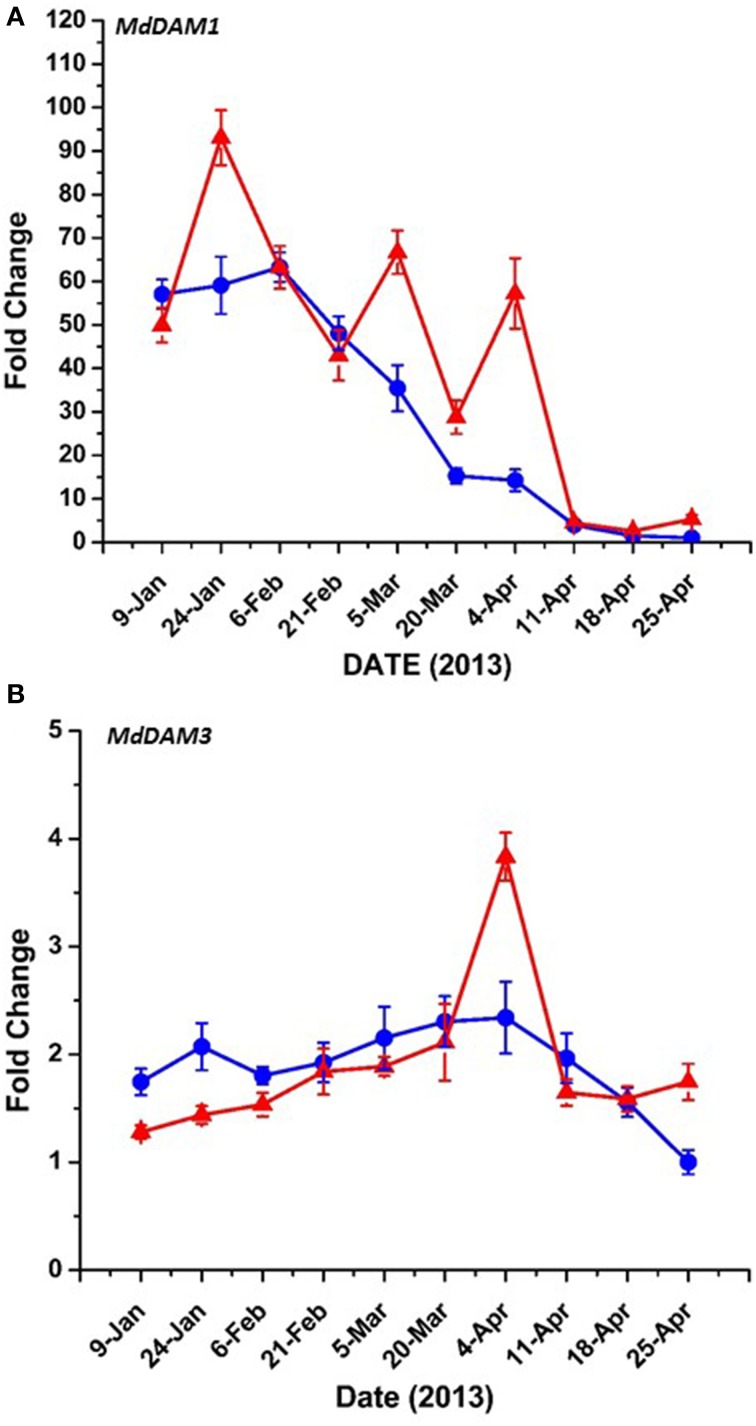
***MdDAM* transcript accumulation kinetics from bud tissue during winter and early spring vary between genes. (A)** *MdDAM1*. **(B)** *MdDAM3*. *MdDAM2* was not detected. Blue circles: non-transformed M.26; Red triangles: Line T166.

Bioinformatic analysis identified four putative *DAM* genes in the apple genome as listed in Genomic Database for the Rosaceae (GDR) (http://www.rosaceae.org) (Figure S2). Three (MDP0000527190, MDP0000322567, MDP0000259294,) bear high similarity to reported *DAM* genes in pear (Saito et al., 2013), and an additional predicted gene (MDP0000209705) was also considered due to its similarity to the other putative *DAM* genes. We have annotated three of these genes thusly: MDP0000322567 = *MdDAM1* (KP164996), MDP0000259294 = *MdDAM2* (KP164997), and MDP0000209705 = *MdDAM3* (KP164998). Despite the use of several primers (Table S1) and protocol adjustments no measurable expression was observed for MDP0000527190 in either bark or bud tissues. As such, it was not assigned an *MdDAM* designation. Seasonal expression of the other three putative *DAM* genes in bark tissues collected from trees of M.26 and T166 are presented in Figure 6. Similar patterns of expression were observed for *MdDAM1* in both genotypes (Figure 6A). Levels of expression rose in the fall, reached a maximum in November/December and then declined reaching a minimum in April. In contrast, levels of expression for *MdDAM2* began to increase in mid-summer, reached a maximum in September/October and declined and remained low throughout the winter and spring months (Figure 6B). Notably, *MdDAM2* expression was higher in January–March in the T166 trees, and a brief rapid increase in the expression level of *MdDAM2* was observed during April/May (Figure 6B). The pattern of *MdDAM3* was similar in both genotypes except for a single spike in expression in the T166 trees during April/May (Figure 6C). This was similar to the spike in expression observed for *MdDAM2* (Figure 6B). In bud tissues, expression of *MdDAM1* and *MdDAM3* were the only *DAM* genes for which products could be obtained by RT-qPCR (Figure 7). In contrast to bark tissues, where expression levels of *MdDAM1* were similar, the level of expression differed significantly in bud tissues (Figure 7A) collected from the transgenic (T166) and non-transformed (M.26) trees. Overall the expression level of *MdDAM1* was higher during the winter months and then declined during the spring in both genotypes. In buds of T166 plants, however, several spikes in expression were observed in early spring. A similar trend was observed for the level of expression of *MdDAM3* in bud tissues of both genotypes, however, in the case of buds from T166 trees only a single spike in expression was observed (Figure 7B).

**Figure 8:**
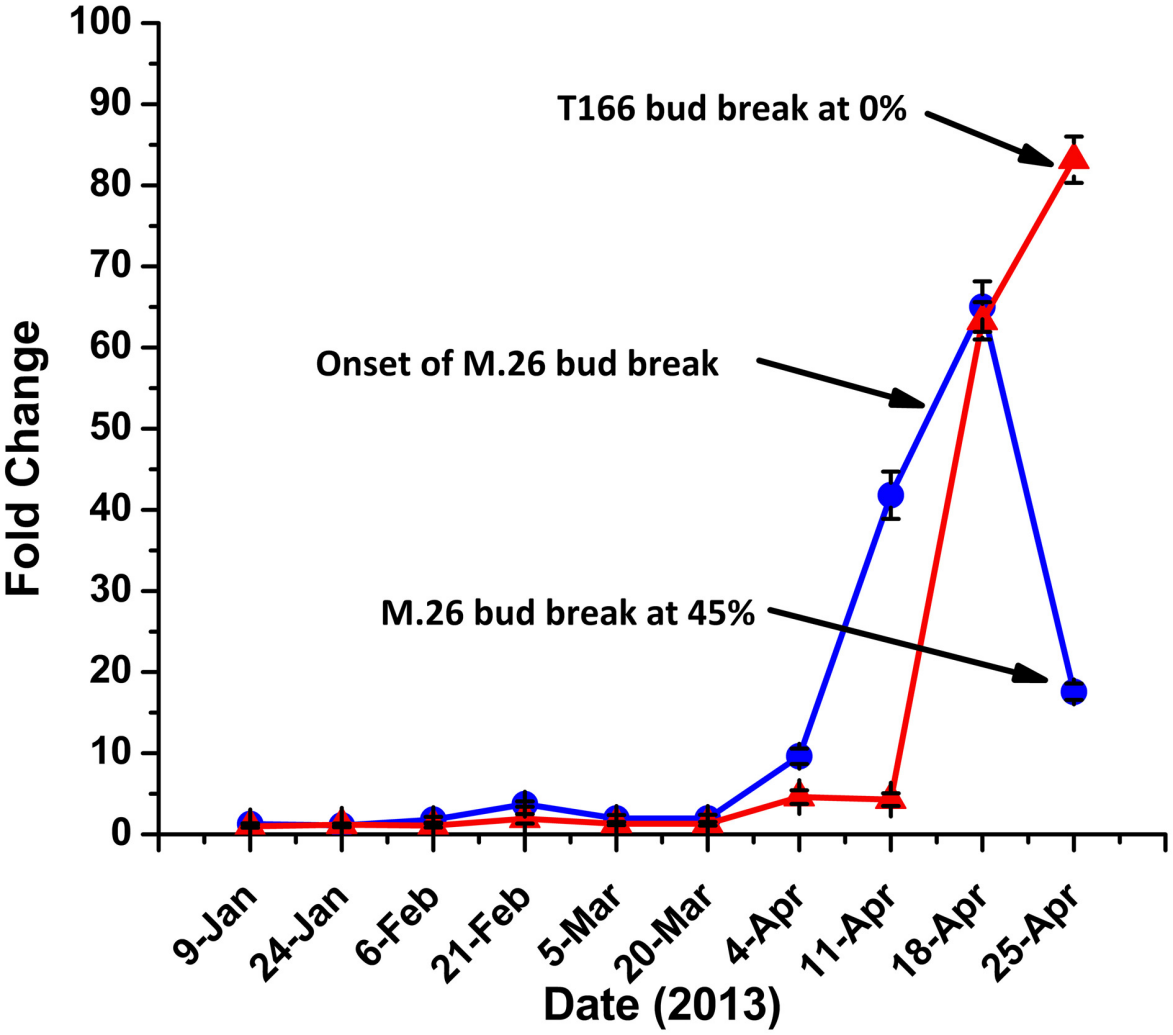
***MdEBB1* transcript accumulation kinetics in winter through spring correlate with delayed bud break in T166**. Blue circles: non-transformed M.26; Red triangles: Line T166.

Yordanov et al. (2014) recently demonstrated the functional role of *EEB1*, a putative APETALA2/Ethylene responsive transcription factor, in determining the time of bud break in poplar (*Populus tremuloides*). BLAST analysis of the apple genome revealed two homologs, MDP0000827400 and MDP0000123172, of the poplar *EBB1* gene (Figure S3). Expression of either gene was not observed in bark tissues (data not shown), however, expression of MDP0000827400 was observed in bud tissues, exhibiting a pattern of expression that could be associated with the timing of bud break observed in the two genotypes (Figure 8). As such, we define MDP0000827400 = *MdEBB1* (KP164995). Induction of *MdEBB1* began earlier in the non-transformed M.26 trees, as did the occurrence of bud break. In contrast, expression in buds of T166 trees was induced about 2 weeks later and rose to higher relative levels. This delay was in agreement with the delay in bud break observed in the T166 trees. The onset of bud break in M.26 trees occurred just prior to peak expression of *MdEBB1*, however, a similar connection could not be determined in T166 trees due to a limited collection of buds.

### DELLA gene expression and growth

**Figure 9:**
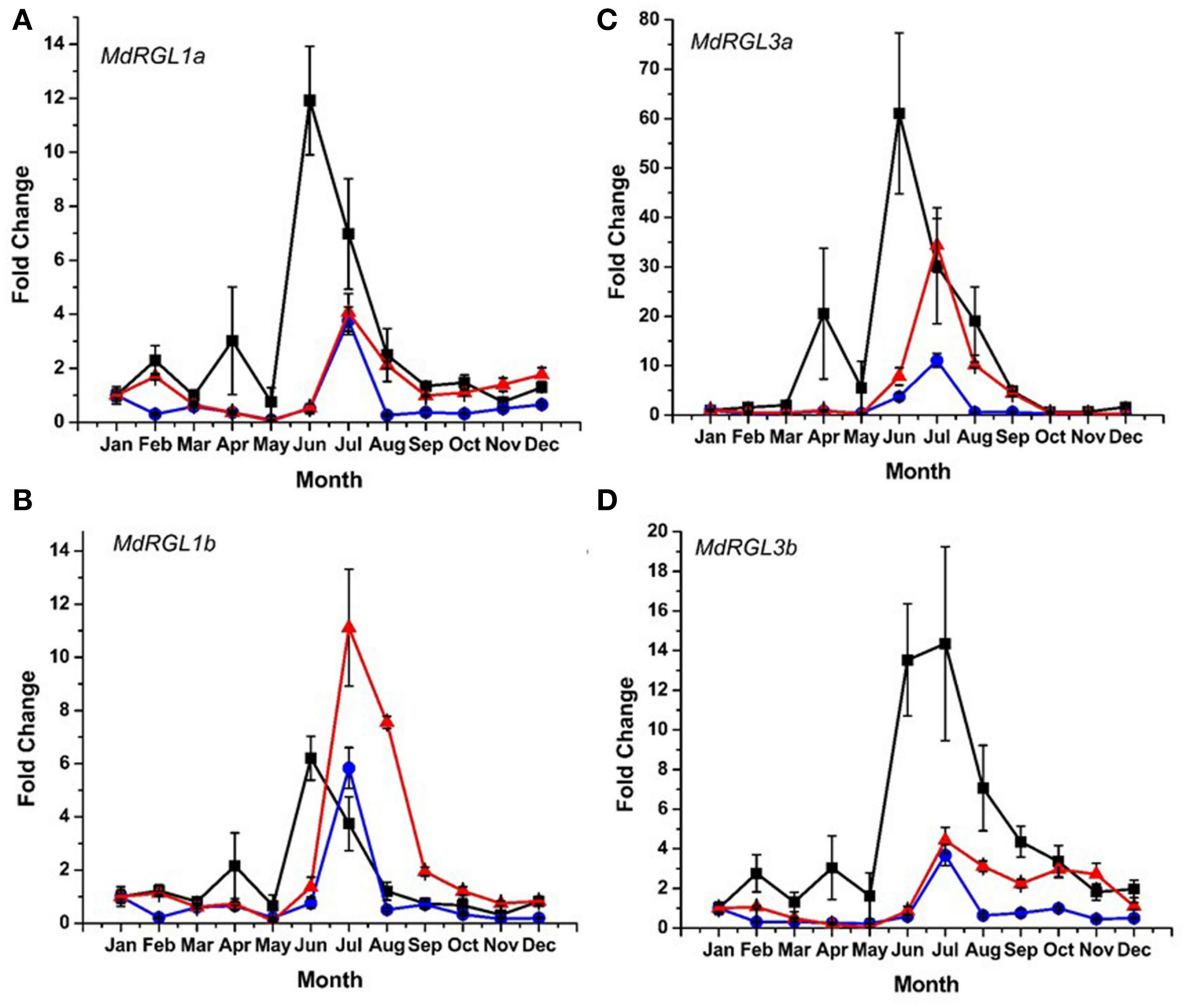
**Seasonal *MdRGL* (*MdDELLA*) transcript accumulation kinetics from bark tissue of three apple genotypes vary between genes and genotypes**. **(A)** *MdRGL1a*. **(B)** *MdRGL1b*. **(C)** *MdRGL3a*. **(D)** *MdRGL3b*. Blue circles: non-transformed M.26; Red triangles: Line T166; Black squares: “CrimsonCrisp.”

DELLA protein abundance is inversely related to the amount of bioactive forms of gibberellic acid (GA) (Achard and Genschik, 2009). Since distinct differences in seasonal patterns and overall levels of growth were observed in T166 vs. M.26 trees, an analysis of *RGL* gene expression was conducted in the two genotypes. The pattern of *RGL* gene expression was also examined in a scion genotype (“CrimsonCrisp”) for comparative purposes. Six *DELLA* genes have been identified in the apple genome (Foster et al., 2007); Figure S4. Using various primer sets (Table S1), the seasonal pattern of expression of four of the apple *DELLA* genes (*MdRGL1a*, *1b*, *3a*, and *3b*) could be characterized. Expression of *MdRGL2a* and *2b* could not be discerned. In all three genotypes, the highest level of expression of the four *DELLA* genes was observed during the summer months of July–August (Figure 9). Except for *MdRGL1b* the highest level of expression was observed in the scion genotype, “CrimsonCrisp.” The exception was *MdRGL1b* where T166 trees exhibited the highest fold change. In comparing just the non-transformed (M.26) genotype with the transgenic (T166) line, the transcript abundance of the four *RGL* genes was higher in the T166 samples than in the M.26 samples. An extended period of elevation for *MdRGL3b*, lasting through the fall months (September–November) was observed in T166 trees.

### Promoter analyses

**Figure 10:**
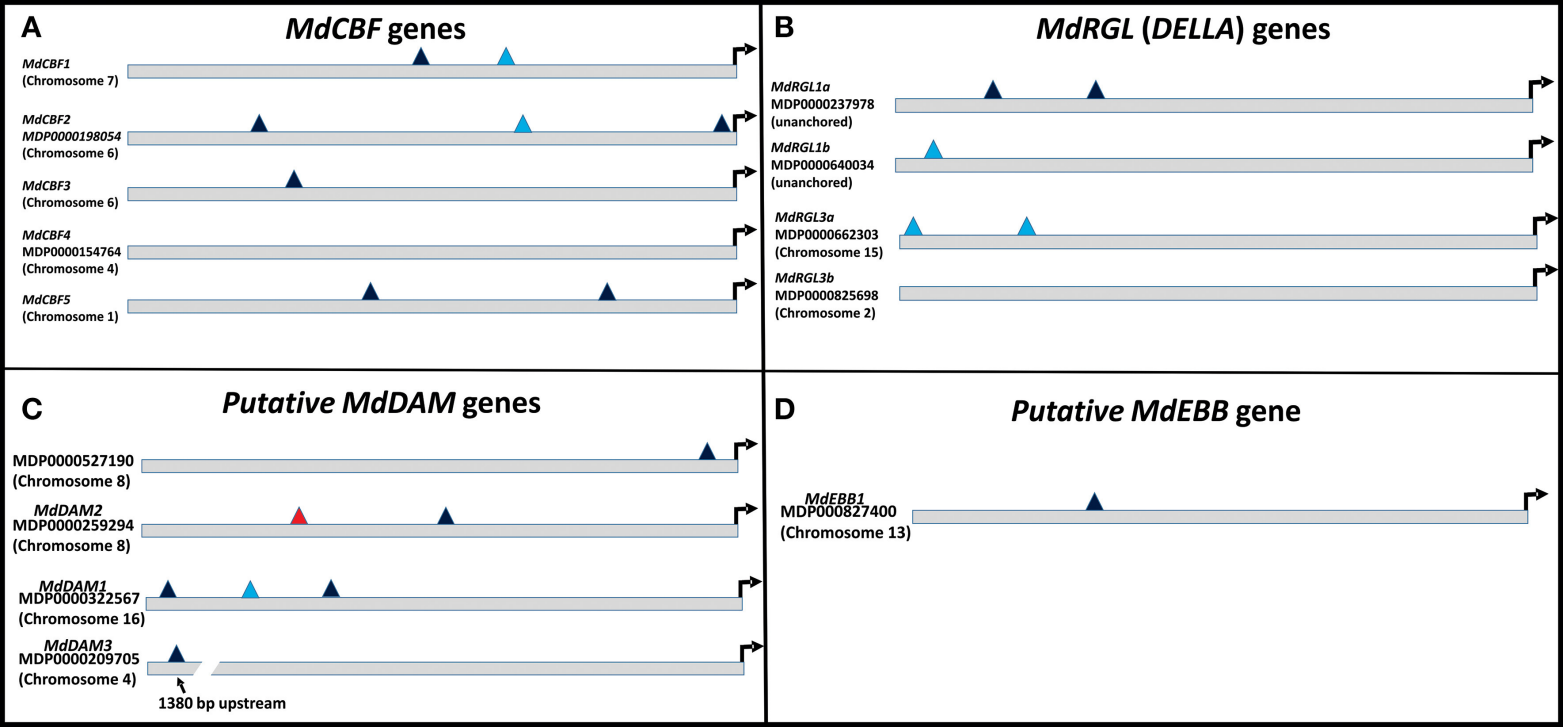
**Schematic of C-repeats present in the 1000 bp upstream of the translational start sites of the genes examined in this report**. C-repeats are binding sites for *CBF* transcription factors. **(A)** *MdCBF* genes. **(B)** *MdRGL* genes. **(C)** *MdDAM* genes. **(D)** *MdEBB1* gene. Dark blue triangles: canonical dicot C-repeats; light blue triangles: monocot C-repeats: red triangles: monocot DRE binding site (nearly identical to C-repeat). Arrow denotes translational start site.

Several phenotypic characteristics were altered in T166 trees, and these changes were, to some degree, reflected in differences in expression of candidate genes associated with cold hardiness, dormancy, bud break, and growth. Therefore, 1000 bp upstream to the start codon of the examined genes were subjected to a bioinformatics analysis using various web-based regulatory element search tools. The presence or absence of the C-repeat (LTRE, Low Temperature Response Element) in these genes was of particular interest, since it could potentially help to explain how overexpression of CBF could lead to changes in these other processes. Results of for the presence of C-repeat elements are presented in Figure 10 and a more detailed and complete analysis is presented in Table S2. The canonical dicot C-repeat element is present in four of the five *MdCBF* genes, suggesting some degree of self- or cross-regulation. Each of the three putative *MdDAM* genes had at least one C-repeat element, with *MdDAM1* having three, one of which represented a C-repeat typical of monocots. Additionally, the C-repeat present in *MdDAM3* was approximately 1500 bp upstream from the coding region. A C-repeat element was also observed in the promoter region of *MdEBB1*, which was more highly expressed in T166 buds, and *MdRGL1a and b* and *MdRGL3a*.

Additional regulatory elements were also observed (Table S2). These included dehydration related elements (Abscissic Acid Response Elements, ABREs, and sites associated with certain MYB transcription factors), light-related elements (PIF, Evening Element, GATA and circadian rhythm) and flowering-related elements (agamous and certain RAV1 transcription factors). *MdCBF* genes were also examined for the presence of ICEr1 (MYC core), ICEr2, and CAMTA binding sites (Table S2).

## Discussion

Cold acclimation, dormancy, and the timing of bud break are parameters that play a critical role in the life cycle of temperate woody plants (see Reviews by Cooke et al., 2012; Rios et al., 2014; Wisniewski et al., 2014). Research by Wisniewski et al. (2011) has documented that overexpression of a peach CBF gene (*PpCBF1*) in a rootstock variety (M.26) of apple resulted in an increase in both non-acclimated and acclimated cold hardiness, early induction of budset and dormancy stimulated by exposure to short day length, and growth inhibition. These observations were further confirmed by 3 years of field observations of trees of the transgenic line (T166) and the non-transformed parent line (M.26) and also revealed that, in addition to early leaf senescence and dormancy, T166 also exhibited delayed bud break in the spring (Artlip et al., 2014). These findings were further confirmed by a year of extra data presented in the current study (Figures 1–4). Thus, these plants may serve as a useful model for investigating how the regulation of these various life-cycle parameters are integrated. The objective of the present study was to document the expression of all of the apple *CBF* genes in response to cold acclimating conditions and to compare the expression of various genes that have been reported to play a major role in dormancy, bud break, and growth in the T166 and M.26 genotypes. This was done in an attempt to better understand how the regulation of these various life-cycle parameters are integrated.

### CBF gene expression

Five *CBF* genes have been identified in the genome of apple (Zhuang et al., 2011; Wisniewski et al., 2014). The expression of only three (*MdCBF1, 2*, and *4*) of the five *CBF* genes could be discerned in leaf tissues collected from M.26 trees subjected to cold acclimating conditions (4°C, 8/16 h light/dark photoperiod) suggesting that not all the *CBF* genes in apple are responsive to cold acclimating conditions or that expression of individual *CBF* genes may be tissue specific (Figure 5). The strongest induction occurred in *MdCBF4* which was induced after 2 h and peaked at 24 h. The next highest level of induction was observed in *MdCBF4*. An earlier report (Wisniewski et al., 2011) indicated that the normal expression pattern of *MdCBF1* and *2* were not altered by the ectopic expression of *PpCBF1*, so this aspect was not further investigated in the present study.

Differential expression in different tissues and differential levels of transcript accumulation of *CBF* genes has been commonly observed (Wisniewski et al., 2014). In detailed studies of grape, Xiao et al. (2008) reported that *VrCBF4* exhibited induced expression for 0.5 h to 2 days in both younger and older leaves, while *VrCBF1, 2*, and *3* accumulated only in young tissues with maximum expression occurring at 30 min, 8 h, and 5 days, respectively (Xiao et al., 2006). *CBF* genes have also been demonstrated to respond to varying degrees to different abiotic stresses. Overexpression of *AtCBF1, 2* or *3* enhanced cold tolerance and also drought and salt tolerance (Mizoi et al., 2012). Mantri et al. (2012) have noted a significant overlap between drought- and cold-stress induced transcriptomes. Epigenetic regulation may also play a role in the expression of specific transcription factors (Rios et al., 2014). Therefore, it would not be unexpected to find the different levels of expression and perhaps tissue-specific expression noted in the present study.

The impact of CBF expression on other plant developmental processes has also been noted, reduced growth and late flowering being two notable effects (Gilmour et al., 1998; Lazaro et al., 2012). The involvement of *CBF* genes in seed dormancy, via their induction of genes that lower GA expression has also been reported (Kendall et al., 2011). Many of these same features have been observed in the T166 transgenic apple line investigated in the present study. In this regard, it is interesting to note that all of the *MdCBF* genes, except *MdCBF4*, and other transcription factors, except *MdRGL3b*, examined in the present study contain C-repeat elements in the promoter regions of their genes (Figure 10). Although the integrated regulation of cold acclimation, dormancy, and growth will inevitably be shown to be complex, this is one way in which self-regulation of *CBF* gene expression, and cross-regulation amongst *CBF* genes and between *CBF* genes and other key regulatory transcription factors could occur and be integrated. Evidence for this level of cross-talk and regulation has yet to be conclusively demonstrated in our transgenic apple but the current study lays the foundation for future experiments.

### Dormancy and bud break-related gene expression

*Dormancy Associated MADS-box* (*DAM*) genes have been reported to be directly associated with the regulation of dormancy onset and release in peach (Bielenberg et al., 2008; Li et al., 2009; Jimenez et al., 2010), pear (Saito et al., 2013), and apricot (Sasaki et al., 2011) trees, as well the herbaceous plant, leafy spurge (*Euphorbia esula*) (Horvath et al., 2010), and indirectly in apple (Falavigna et al., 2014). T166 trees exhibit early induction of dormancy and leaf senescence in the summer\autumn and delayed bud break in the spring. This altered phenology was initially reported in greenhouse-grown plants (Wisniewski et al., 2011), then in field-grown plants (Artlip et al., 2014), and further confirmed in the current study (Figures 1, 2). Both previous studies postulated a role for *DAM* genes in the altered phenotype and so they were further investigated in the current study.

The putative apple *DAM* genes, *MdDAM1*, *MdDAM2*, and *MdDAM3*, defined in the present study, have high similarity to the *Pyrus pyrifolia MADS13* genes (Saito et al., 2013), and other *DAM* genes reported in apricot and peach, however, phylogenetic relationships and analogous functions still need to be determined. While a complete set (a full year) of data is available on the expression of three of the putative apple genes (Figure 6) in bark tissues only a partial set (late winter through spring) of data are available for *DAM* gene expression in vegetative apple buds (Figure 7). In bark tissues, the pattern of seasonal expression of all three apple *DAM* genes (*MdDAM1*, *MdDAM2*, *MdDAM3*) were similar in both genotypes, however, a sharp spike in expression was noted in the expression of *MdDAM2* and *MdDAM3* in T166 trees in April/May. This suggests that high levels of expression of these genes may inhibit bud break as trees would have fulfilled their chilling requirement by this time and presumably would have been ecodormant rather than endodormant. In this regard, expression of *MdDAM2* was significantly greater in T166 trees in January through March and may have played a more direct role in dormancy release (transition from endodormancy to ecodormancy). Expression of only two (*MdDAM1* and *MdDAM3*) could be detected in apple vegetative buds (Figure 7). While the overall pattern of expression of these two *DAM* genes was again similar in both genotypes, a single sharp spike was observed in the T166 buds in early April and several spikes in expression were observed in *MdDAM1* during January through April in T166 buds. Interestingly, expression levels for *MdDAM1* did not differ at all in bark tissues. This suggests that this *DAM* gene may play a more important role in the regulation of bud dormancy/bud break while the other *DAM* genes may play a more important role in the regulation of cambial dormancy (collection of bark tissues from current year shoots would have included sampling of phloem and cambial tissues).

A variety of seasonal patterns of *DAM* gene expression have been noted in other woody plant species (Li et al., 2009; Sasaki et al., 2011; Saito et al., 2013) as was observed in the present study. In peach, Li et al. (2009) reported that *DAM1, 2*, and *4* were the most likely candidates for control of seasonal elongation cessation and bud formation, while Jimenez et al. (2010) reported that *DAM5* and *DAM6* were negatively correlated with chill hour accumulation and the rate of bud break. In apricot, *PmDAM4* and *PmDAM6* have been suggested as the primary candidates regulating endodormancy and chilling requirement (Sasaki et al., 2011), and in pear all three reported *DAM* genes (*PpMADS13-1, 13-2*, and *13-3*) were reported to be associated with endodormancy establishment and release and were impacted by the application of dormancy-releasing agents (Saito et al., 2013). In the latter study *PpMADS13-1* was reported to be specifically associated with dormancy release. In apple, the seasonal pattern of expression of *MdDAM1* has been reported to exhibit a dormancy-related expression in three different cultivars (Falavigna et al., 2014), and to differ in the level of expression in low and high chill varieties. In the current study in apple, three *DAM* genes (*MdDAM1*, *MdDAM2*, *MdDAM3*) exhibited patterns of expression that could be associated with dormancy onset and release. However, only the expression pattern of *MdDAM2* and *MdDAM3* differed between the two genotypes (T166 and M.26) making them the most likely candidates for fine tuning the regulation of dormancy in apple, and perhaps more specifically cambial dormancy. In buds, only the expression of two *DAM* genes (*MdDAM1* and *MdDAM3*) could be detected, with both genes showing a different pattern of expression in T166 vs. M.26 plants that may have impacted bud break. Collectively, our results in apple buds confirm the results obtained by Falavigna et al. (2014) and provide new information on the potential role of *DAM* genes in cambial dormancy. Importantly, as noted with the *CBF* genes, all the *DAM* genes identified in the current study possess C-repeat elements in their promoters, although the C-repeat element in *MdDAM3* is somewhat removed (1380 bp) from the coding region of the gene (Figure 10).

It is important to note that release from dormancy and time to bud break are two different phenological events and may have different modes of genetic regulation. While release from dormancy is associated with the accumulation of chilling hours, time to bud break after dormancy release is associated with the accumulation of heat units. Yordanov et al. (2014) recently reported that overexpression of a putative poplar APETALA2/Ethylene responsive factor transcription factor in poplar caused early bud-flush, whereas down-regulation delayed budbreak. The gene was highly expressed in actively growing apices, and was undetectable in dormant buds. Two *EBB* like genes were identified in the current study, however, expression of only one of them (*MdEBB1*), could be detected in vegetative apple buds. No expression was observed in bark tissues (data not shown). Expression of *MdEBB1*, as in poplar, was low to non-detectable in dormant buds and began to rapidly increase just prior to the onset of budbreak (Figure 8). Notably, levels of *MdEBB1* began to increase earlier in the non-transformed M.26 genotype than in the transgenic T166 genotype. The M.26 trees also exhibited earlier bud break than T166 trees (Figures 1, 2), hence *MdEBB1* should be considered a strong candidate, in addition to *DAM* genes, for regulating bud break in apple trees. As with other genes investigated in this study, *MdEBB1*, also possesses a C-repeat element in its promoter region. This is the first association of this putative class of transcription factors with the regulation of dormancy and/or bud break in apple.

### RGL (DELLA protein) gene expression

T166 trees exhibit reduced overall growth, as well as a reduced number of lateral branches compared to non-transformed M.26 trees (Figures 3, 4). Growth inhibition due to over-expression of native and foreign *CBF* genes has been reported in *Arabidopsis* (Jaglo-Ottosen et al., 1998; Kasuga et al., 1999; Gilmour et al., 2000; Welling and Palva, 2008). Over-expression of two different native *Eucalyptus CBF* genes in *Eucalyptus* and the native *VvCBF4* gene in grape (*Vitus vinifera*) also resulted in reduced growth (Navarro et al., 2011; Tillett et al., 2012).

The reduced-growth phenotype in Line T166 and other systems is most likely caused directly or indirectly by a reduction in the level of bio-active giberellic acid (GA). Achard et al. (2008), Suo et al. (2012) and Niu et al. (2014) have all reported changes in the expression levels of GA-biosynthetic and GA-deactivating genes in plants over-expressing *CBF* genes (*Arabidopsis thaliana*, soybean (*Glycine max*) and tobacco (*Nicotiana tabacum*), respectively), with Suo et al. (2012) and Niu et al. (2014) noting decreased GA levels in such plants. The reduction in bioactive GA levels have been attributed to increases in GA2ox (GA deactivating) enzymes (Achard et al., 2008; Suo et al., 2012) or to a decrease in the geranylgeranyl diphosphate precursor to GAs (Niu et al., 2014). Application of exogenous bio-active GA was shown to overcome the dwarfism associated with *CBF* gene over-expression (Achard et al., 2008; Suo et al., 2012). Yang et al. (2013) indicate that GA_3_ is primary bioactive GA in apple vegetative tissues. Examination of GA_3_ levels along with expression data on GA-biosynthetic and GA-deactivating genes in Line T166 and non-transformed M.26, however, were not conducted in the present study.

An additional cause of reduced growth levels in Line T166 may stem from an up-regulation of DELLA proteins which normally repress GA responses. Achard et al. (2008) demonstrated that over-expression of *AtCBF1*in *Arabidopsis thaliana* up-regulates *RGL3* which leads to enhanced levels of DELLA protein(s). GAs normally mediate the turnover of DELLAs by direct and indirect means (Achard and Genschik, 2009), hence a reduction in bio-active GAs also leads to enhanced levels of DELLA proteins.

There are six apple *DELLA* genes, *MdRGL1a*, *1b*, *2a*, *2b*, *3a*, and *3b*, with each *a*-*b* pair highly related to each other and having nearly identical expression patterns (Foster et al., 2007). The seasonal expression of four *MdRGL* genes (*1a*, *1b*, *3a*, and *3b*) was examined in bark tissues (Figure 9). The overall pattern of seasonal expression for these genes was similar in T166 and non-transformed M.26 trees. All four genes exhibited increased expression during the summer, with peaks occurring in July. Artlip et al. (2014) reported that current year shoot growth in both these genotypes dramatically slows in July–August. This slowing down in growth coincides with a peak in *MdRGL* expression in July (Figure 7). Foster et al. (2007) noted that shoots with arrested growth had higher *MdRGL* expression levels than actively growing shoots. In general, higher fold increases were observed for these genes in T166 bark tissues than in M.26 bark tissues. This was especially apparent for *MdRGL1a* during the period of late summer into early winter, *MdRGL1b* during summer and early autumn, *MdRGL3a* during summer and early autumn, and *MdRGL3b* during summer into early winter. These data indicate that *MdRGL* gene products may play a role in the reduced growth in T166 trees, relative to non-transformed M.26 trees.

Notably, expression of the *RGL* genes in the scion cultivar “CrimsonCrisp” was much greater than in the transgenic and non-transgenic M.26 trees, except for *MdRGL1b* (Figure 7). Achard and Genschik (2009) have suggested that levels of DELLA proteins are coupled with GA levels in order to maintain GA homeostasis. It is possible that scion cultivars such as “CrimsonCrisp” synthesize higher levels of GA compared to root-stock trees, and require greater levels of DELLA proteins to maintain GA homeostasis. This premise is supported by the observation that own-rooted, “Royal Gala” trees (“very vigorous” growth habit) have greater levels of root-sourced GA than “Royal Gala” on root stocks varying in their vigor (van Hooijdonk et al., 2011).

Potential regulation of the examined *MdRGL* genes by CBF is possible, as *MdRGL1a* has two canonical dicot C-repeats, while *MdRGL1b* and *MdRGL3a* have monocot-related C-repeats. (Figure 10; Table S2). These genes all show greater fold changes in Line T166 compared to non-transformed M.26. *MdRGL1b*, however, also displayed similar pattern of expression, despite having no C-repeat (Figure 10; Table S2). This suggests that *CBF* genes represent only one method of regulating *RGL* gene expression. Indeed, various promoter elements related to light and dehydration are present in all of the *MdRGL* genes (Table S2). The similar pattern of expression for all the examined *MdRGL* genes in apple (Figure 9) also supports the premise stated by Foster et al. (2007), of overlapping or perhaps redundant functions between *MdRGL* gene products.

### Promoter analyses

The upstream 1000 bp prior to the translational start sites of the investigated genes were examined for the presence of various promoter elements (Table S2). In addition to the Low Temperature Response Element (LTRE or C-repeat), other promoter elements were identified, including ABA and dehydration responsive (ABRE, MYB1A, and MYB2), light or circadian rhythm (GATA, PIF, Evening Element, CircadianLHC) and bud or floral development (RAV or AGAMOUS). All of the promoter regions of the investigated genes had these elements, suggesting multiple regulatory possibilities. The presence of dehydration- or ABA-responsive elements in growth repressing *DAM* or *DELLA* genes is consistent with a need to repress growth during periods of water-limitation. Regulatory elements associated with light or circadian rhythm are also consistent with the need to integrate appropriate light cues for growth. An analysis to determine the presence of regulatory elements frequently found in *CBF* genes such as MYC (core of the ICEr1 binding site) and TGGAGGC (ICEr2 binding site) (Zarka et al., 2003), CAMTA (Calmodulin binding Transcription Activator) and Common Motifs (CMs) (Doherty et al., 2009) was also conducted. In contrast to *Arabidopsis thaliana*, few of these motifs were consistently found in the promoter region of the *MdCBF* genes. A CAMTA motif was found in *MdCBF1*, implying regulation by Ca^2+^ (Doherty et al., 2009). *MdCBF4* had an ICEr1-like motif but no ICEr2 motif, while *MdCBF5* had an ICEr2 motif but no recognizable ICEr1 motif.

## Conclusions

**Figure 11:**
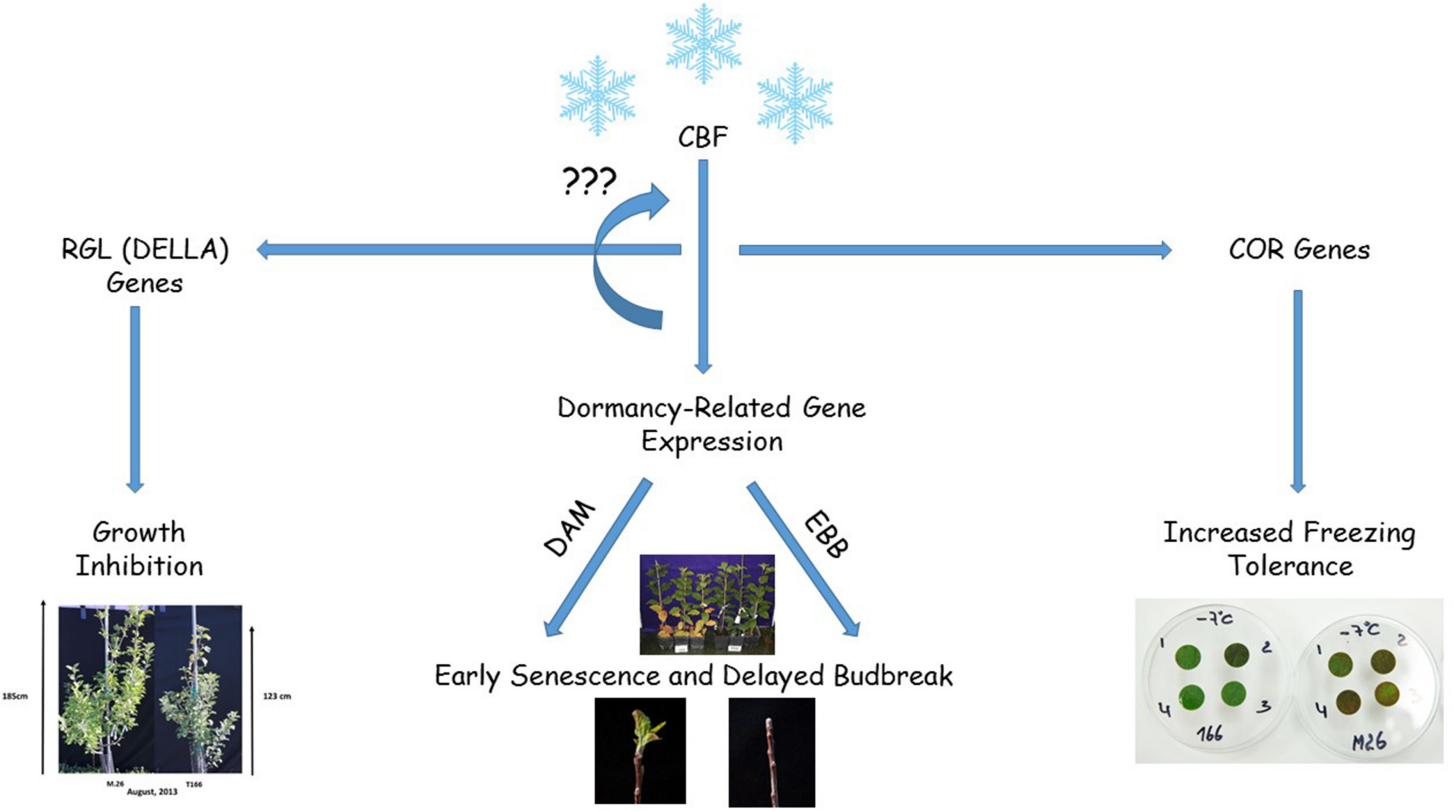
**Diagram summarizing the results obtained in the current study where a peach *CBF* (*PpCBF1*) was overexpressed in an apple (*Malus* × *domestica*) rootstock variety, M.26**. Overxpression of the *CBF* gene (*PpCBF1*) results in altered expression of apple dormancy-related *genes* (*MdDAM_x_* and *MdEBB_x_*) resulting in early senescence in the fall and delayed budbreak in the spring. Overexpression of the *CBF* gene also leads to altered expression of *RGL* genes (*MdRGL_x_*) which results in growth inhibition. *CBF* overexpression also induces the expression of *COR* genes which results in increased freezing tolerance (Wisniewski et al., 2011). The regulation of the various genes and other *CBF* genes may be due to the presence of C-repeat motifs present in the promoter region of the studied genes.

The timing of cold acclimation and deacclimation, the onset and release from dormancy, and the timing of bud break and onset of growth are overlapping, integrated processes that play an essential role in the life cycle of woody plants and their adaptation to the external environment. Overexpression of a peach *CBF* (*PpCBF1*) in a rootstock variety of apple (M.26) alters many of these parameters, exhibiting increased cold hardiness, early cessation of growth and leaf senescence, delayed bud break in the spring, growth inhibition, and increased sensitivity to short photoperiod with respect to the onset of dormancy. In the current study, several transcription factor genes that have been reported to regulate one or more of these processes was examined. Results indicated that expression of several of these key genes, including *MdDAM*, *MdRGL*, and *MdEBB* was altered in transgenic T166 trees relative to non-transformed M.26 trees. In particular, several *MdDAM* genes, associated with the dormancy-cycle in other species of woody plants in the Rosaceae, exhibited different patterns of expression in the T166 vs. M.26 trees. Additionally, for the first time a putative APETALA2/Ethylene responsive factor transcription factor, originally described in poplar and shown to regulate the timing of bud break, was shown to be associated with the timing of bud break in apple. Since the overexpression of *PpCBF1* in apple results in a dramatic alteration in cold acclimation, dormancy, and growth, this transgenic line (T166 and others) may represent a useful model for studying the integration of these seasonal life-cycle parameters (Figure 11).

### Conflict of interest statement

The authors declare that the research was conducted in the absence of any commercial or financial relationships that could be construed as a potential conflict of interest.

## Supplementary material

Figure S1 **Alignment of conceptual MdCBF1-5 and PpCBF1 amino acid sequences**. Light red residues indicate different residues between the sequences. The alignment was performed with CLUSTALW (Thompson et al., 1994).

Figure S2 **Alignment of conceptual MdDAM, peach DAM5 and DAM6, and pear PpMADS13-1, 13-2 and 13-3 amino acid sequences**. Light red residues indicate different residues between the sequences. The alignment was performed with CLUSTALW (Thompson et al., 1994).

Figure S3 **Alignment of conceptual MdEBB and poplar EBB1 amino acid sequences**. Light red residues indicate different residues between the sequences. The alignment was performed with CLUSTALW (Thompson et al., 1994).

Figure S4 **Alignment of conceptual MdRGL (DELLA) amino acid sequences**. Light red residues indicate different residues between the sequences. The alignment was performed with CLUSTALW (Thompson et al., 1994).
