# Supplementary material for: Overexpression of a peach CBF gene in apple: a model for understanding the integration of growth, dormancy, and cold hardiness in woody plants
Source: Front Plant Sci. 2015 Feb 27;6:85. doi: 10.3389/fpls.2015.00085 (PMC4343015; doi:10.3389/fpls.2015.00085)
Supplement: Figure S1 — Alignment of conceptual MdCBF1-5 and PpCBF1 amino acid sequences. Light red residues indicate different residues between the sequences. The alignment was performed with CLUSTALW (Thompson et al., 1994). [file DataSheet1.ZIP › Table 1.DOCX]

Supplementary Table 1. Primers used for RT-qPCR

| **Gene** | **For (5' - 3')** | **Rev (5' - 3')** |
| --- | --- | --- |
| **MdCBF1** | **cagatatggcggaaggaa** | **aaaaactccataagggcac** |
| **MdCBF2** | **tctccgcctcactcttca** | **gaaagcgtccgaaagtt** |
| **MdCBF4** | **actgggatgatatgggaa** | **gtgtcctttagggatgat** |
| **MdDAM2** | **tcaccaccagctccaaca** | **agcacaagccaaaagtcaaa** |
| **MdDAM3** | **gagggtcagtcatcagag** | **gcagcacatcaaacatatca** |
| **MdDAM1** | **accacaaatgtcaccacct** | **ccaccaaatactcaaaacgcaa** |
| **DELLA 1A** | **agaacgacgggtgtatga** | **cacccaaaaaaaatggaaccga** |
| **DELLA 1B** | **ctcattgccacctcggctt** | **aaccccaccaccatcacca** |
| **DELLA 3A** | **cacaatcaaccaccaaac** | **gaagtaacacaaagagcaag** |
| **DELLA 3B** | **gaagccacggcaaaccaa** | **cattaaggtagaacacagac** |
| **MdEBB** | **tgaggttatgggtgagtttg** | **atgggaacattttgcagg** |
| **EF** | **gacattgccctgtggaagtt** | **ggtctgaccatccttggaaa** |
| **26s** | **gcagccaagccttcatagcg** | **gtgcgaatcaacggttcctc** |
| **MdCBF3** | **ctctccacctcactcttca** | **gggtcaacgaacgaaagc** |
| **MdCBF3** | **ctctctccacctcactcttca** | **caacgaacgaaagcgtcc** |
| **MdCBF3** | **tctgctctctccacctca** | **gaaagcgtcctccggata** |
| **MdCBF5** | **ttttatggatgaggaagcg** | **attgtaatgaggtggagg** |
| **MdCBF5** | **ttatggatgaggaagcggt** | **gtaatgaggtggaggcag** |
| **MdCBF5** | **tatggatgaggaagcggt** | **gtaatgaggtggaggcag** |

Table S1 Legend:

| Gene names include Genome Database for Rosaceae predicted transcript accession numbers. Bold face denotes primer pairs used to generate RT-qPCR results, while regular face denotes primer pairs that were deemed unacceptable. *EF2*, *transla­tion elongation factor; 26S, 26S rRNA*. |
| --- |
