# Supplementary material for: Overexpression of a peach CBF gene in apple: a model for understanding the integration of growth, dormancy, and cold hardiness in woody plants
Source: Front Plant Sci. 2015 Feb 27;6:85. doi: 10.3389/fpls.2015.00085 (PMC4343015; doi:10.3389/fpls.2015.00085)
Supplement: Figure S1 — Alignment of conceptual MdCBF1-5 and PpCBF1 amino acid sequences. Light red residues indicate different residues between the sequences. The alignment was performed with CLUSTALW (Thompson et al., 1994). [file DataSheet1.ZIP › Table 2.DOCX]

Supplementary Table 2. Selected promoter elements of *MdCBFs1–5*, *MdRGLs1-3*, putative *MdDAM* and putative *MdEBB* genes.

|  |  |  |  |  |  |
| --- | --- | --- | --- | --- | --- |
| **Gene** | **Motif** | **Position** | **Strand** | **Sequence in Promoter** | **Published Consensus Sequence** |
| **MdCBF1** | **CM1** | **785** | **-** | **GACCACA** | **GACCMCA** |
|  | **CM2 (CAMTA)** | **755** | **+** | **GCGCGT** | **VCGCGB** |
|  | **CM7** | **507** | **-** | **GGGTCAAAG** | **GGGTCAAAG** |
|  | **LTRE** | **501** | **+** | **CCGAC** | **A/GCCGAC** |
|  | **LTRE1HVBLT49** | **636** | **+** | **CCGAAA** | **CCGAAA** |
|  | **MYCCONSENSUSAT** | **592** | **+** | **CACTTG** | **CANNTG** |
|  | **MYCCONSENSUSAT** | **658** | **+** | **CAATTG** | **CANNTG** |
|  | **MYCCONSENSUSAT** | **669** | **+** | **CAGCTG** | **CANNTG** |
|  | **MYCCONSENSUSAT** | **79** | **+** | **CAAATG** | **CANNTG** |
|  | **MYCCONSENSUSAT** | **98** | **+** | **CAAGTG** | **CANNTG** |
|  | **MYCCONSENSUSAT** | **388** | **+** | **CAAGTG** | **CANNTG** |
|  | **MYCCONSENSUSAT** | **437** | **+** | **CAGGTG** | **CANNTG** |
|  | **MYCCONSENSUSAT** | **451** | **+** | **CATCTG** | **CANNTG** |
|  | **ABRE/ G-box** | **337** | **-** | **ACACGTT** | **YACGTGGC** |
|  | **ABRE/ G-box** | **754** | **-** | **GCGCGTT** | **YACGTGGC** |
|  | **MYB1** | **584** | **+** | **AAACCA** | **WAACCA** |
|  | **MYB1** | **279** | **+** | **TAACCA** | **WAACCA** |
|  | **MYB1** | **314** | **+** | **AAACCA** | **WAACCA** |
|  | **MYB2** | **113** | **+** | **TAACTG** | **TAACTG** |
|  | **RAV1** | **379** | **+** | **aatCAACAgcaa** | **NNGCAACAKAWN** |
|  | **RAV1** | **393** | **+** | **gagCAACAaatc** | **NNGCAACAKAWN** |
|  | **RAV1** | **451** | **-** | **catcTGTTGgct** | **NNGCAACAKAWN** |
|  | **RAV1** | **828** | **+** | **aacCAACAcaat** | **NNGCAACAKAWN** |
|  | **AGAMOUS** | **635** | **-** | **TCCGAAAAAGG** | **CCWWWWNNRGH** |
|  | **CIACADIANLELHC** | **831** | **+** | **CAACACAATC** | **CAANNNNATC** |
|  | **CIACADIANLELHC** | **943** | **-** | **GATTCGCTTG** | **CAANNNNATC** |
|  | **CIACADIANLELHC** | **235** | **+** | **CAATATTATC** | **CAANNNNATC** |
|  | **GATA** | **65** | **-** | **GATA** | **GATA** |
|  | **GATA** | **241** | **-** | **GATA** | **GATA** |
|  | **GATA** | **681** | **-** | **GATA** | **GATA** |
|  | **GATA** | **730** | **+** | **GATA** | **GATA** |
|  | **GATA** | **745** | **-** | **GATA** | **GATA** |
|  | **GATA** | **843** | **-** | **GATA** | **GATA** |
|  | **GATA** | **865** | **-** | **GATA** | **GATA** |
|  | **GATA** | **903** | **-** | **GATA** | **GATA** |
|  | **PIF** | **332** | **+** | **aatacaCACGTttaaatt** | **GKRGGMCACGTGRMSWCK** |
|  |  |  |  |  |  |
| **MdCBF2** | **CM3** | **38** | **+** | **AGAGAC** | **AGAGAC** |
|  | **CM5** | **575** | **+** | **CTTAGCTG** | **CTTMGCTG** |
|  | **LTRE** | **227** | **+** | **CCGAC** | **A/GCCGAC** |
|  | **LTRE** | **983** | **+** | **CCGAC** | **A/GCCGAC** |
|  | **LTRE1HVBLT49** | **27** | **+** | **CCGAAA** | **CCGAAA** |
|  | **LTRE1HVBLT49** | **678** | **+** | **CCGAAA** | **CCGAAA** |
|  | **MYCCONSENSUSAT** | **526** | **+** | **CAAGTG** | **CANNTG** |
|  | **MYCCONSENSUSAT** | **596** | **+** | **CACTTG** | **CANNTG** |
|  | **MYCCONSENSUSAT** | **665** | **+** | **CACGTG** | **CANNTG** |
|  | **MYCCONSENSUSAT** | **713** | **+** | **CACGTG** | **CANNTG** |
|  | **MYCCONSENSUSAT** | **798** | **+** | **CACCTG** | **CANNTG** |
|  | **MYCCONSENSUSAT** | **138** | **+** | **CATGTG** | **CANNTG** |
|  | **MYCCONSENSUSAT** | **186** | **+** | **CATATG** | **CANNTG** |
|  | **ABRE/ G-box** | **624** | **+** | **AACGTGC** | **YACGTGGC** |
|  | **ABRE/ G-box** | **665** | **+** | **CACGTGG** | **YACGTGGC** |
|  | **ABRE/ G-box** | **713** | **+** | **CACGTGTC** | **YACGTGGC** |
|  | **MYB1** | **190** | **-** | **TGGTTT** | **WAACCA** |
|  | **MYB1** | **669** | **-** | **TGGTTA** | **WAACCA** |
|  | **MYB1** | **876** | **+** | **AAACCA** | **WAACCA** |
|  | **Evening Element** | **433** | **+** | **AAAATATCT** | **AAAATATCT** |
|  | **PIF** | **615** | **-** | **catcctaACGTGccaatt** | **GKRGGMCACGTGRMSWCK** |
|  | **PIF** | **653** | **+** | **aactgaCACGTggttaac** | **GKRGGMCACGTGRMSWCK** |
|  | **PIF** | **701** | **+** | **tccagccACGTGtcccta** | **GKRGGMCACGTGRMSWCK** |
|  | **GATA** | **72** | **+** | **GATA** | **GATA** |
|  | **GATA** | **135** | **-** | **GATA** | **GATA** |
|  | **GATA** | **180** | **-** | **GATA** | **GATA** |
|  | **GATA** | **285** | **+** | **GATA** | **GATA** |
|  | **GATA** | **437** | **-** | **GATA** | **GATA** |
|  | **RAV1** | **286** | **-** | **tataTGTTGaat** | **NNGCAACAKAWN** |
|  | **RAV1** | **601** | **+** | **acaCAACAcatc** | **NNGCAACAKAWN** |
|  | **RAV1** | **776** | **+** | **gggCAACAtttt** | **NNGCAACAKAWN** |
|  | **RAV1** | **869** | **+** | **aacCAACAgcta** | **NNGCAACAKAWN** |
|  |  |  |  |  |  |
| **MdCBF3** | **LTRE** | **268** | **+** | **ACCGACA** | **A/GCCGAC** |
|  | **MYCCONSENSUSAT** | **521** | **+** | **CAAGTG** | **CANNTG** |
|  | **MYCCONSENSUSAT** | **665** | **+** | **CATGTG** | **CANNTG** |
|  | **MYCCONSENSUSAT** | **705** | **+** | **CAGCTG** | **CANNTG** |
|  | **MYCCONSENSUSAT** | **712** | **+** | **CACGTG** | **CANNTG** |
|  | **MYCCONSENSUSAT** | **813** | **+** | **CACCTG** | **CANNTG** |
|  | **MYCCONSENSUSAT** | **222** | **+** | **CATATG** | **CANNTG** |
|  | **ABREATRD22** | **709** | **-** | **TGCCACGTGT** | **RYACGTGGYR** |
|  | **ABRE-like** | **712** | **+** | **CACGTGTC** | **BACGTGKM** |
|  | **ABRERATCAL** | **620** | **+** | **AACGTGC** | **MACGYGB** |
|  | **MYB1AT** | **2** | **+** | **AAACCA** | **WAACCA** |
|  | **MYB1AT** | **226** | **-** | **TGGTTT** | **WAACCA** |
|  | **MYB2AT** | **384** | **+** | **TAACTG** | **TAACTG** |
|  | **Evening Element-like** | **306** | **-** | **AGATATTTT** | **AAMAATCT** |
|  | **GATABOX** | **555** | **-** | **TATC** | **TATC** |
|  | **GATABOX** | **132** | **+** | **GATA** | **GATA** |
|  | **GATABOX** | **307** | **+** | **GATA** | **GATA** |
|  | **PIF3** | **91** | **+** | **cacactCACGTccaaggg** | **GKRGGMCACGTGRMSWCK** |
|  | **PIF3** | **117** | **+** | **tcaattCACGTcatcgat** | **GKRGGMCACGTGRMSWCK** |
|  | **PIF3** | **612** | **-** | **catcaaaACGTGccaatt** | **GKRGGMCACGTGRMSWCK** |
|  | **PIF3** | **703** | **+** | **agctgcCACGTgtcacta** | **GKRGGMCACGTGRMSWCK** |
|  | **PIF3** | **703** | **-** | **agctgccACGTGtcacta** | **GKRGGMCACGTGRMSWCK** |
|  | **RAV1** | **673** | **+** | **cacCAACAttaa** | **NNGCAACAKAWN** |
|  | **RAV1** | **796** | **+** | **gggCAACAtgtc** | **NNGCAACAKAWN** |
|  |  |  |  |  |  |
| **MdCBF4** | **ICEr1-like** | **119** | **-** | **GGGACAGGTGGCAGA** | **GGACACATGTCAGA** |
|  | **CM6-like** | **399** | **-** | **ACTTCTTA** | **AGATTCTCA** |
|  | **MYCCONSENSUSAT** | **640** | **+** | **CAATTG** | **CANNTG** |
|  | **MYCCONSENSUSAT** | **678** | **+** | **CATGTG** | **CANNTG** |
|  | **MYCCONSENSUSAT** | **41** | **+** | **CATATG** | **CANNTG** |
|  | **MYCCONSENSUSAT** | **123** | **+** | **CACCTG** | **CANNTG** |
|  | **MYCCONSENSUSAT** | **140** | **+** | **CATATG** | **CANNTG** |
|  | **MYCCONSENSUSAT** | **248** | **+** | **CACCTG** | **CANNTG** |
|  | **MYB1AT** | **668** | **-** | **TGGTTT** | **WAACCA** |
|  | **MYB1AT** | **144** | **-** | **TGGTTA** | **WAACCA** |
|  | **MYB1AT** | **309** | **+** | **AAACCA** | **WAACCA** |
|  | **MYB2CONSENSUSAT** | **825** | **-** | **CAGTTA** | **YAACKG** |
|  | **MYB2CONSENSUSAT** | **987** | **+** | **CAACGG** | **YAACKG** |
|  | **MYB2CONSENSUSAT** | **47** | **-** | **CCGTTG** | **YAACKG** |
|  | **MYB2CONSENSUSAT** | **297** | **-** | **CCGTTG** | **YAACKG** |
|  | **RAV1** | **178** | **-** | **cgtgTGTTGgga** | **NNGCAACAKAWN** |
|  | **RAV1** | **212** | **+** | **cagCAACAtttc** | **NNGCAACAKAWN** |
|  | **RAV1** | **491** | **+** | **catCAACAaaaa** | **NNGCAACAKAWN** |
|  | **RAV1** | **506** | **+** | **tgaCAACAgttc** | **NNGCAACAKAWN** |
|  | **RAV1** | **725** | **+** | **acaCAACAcatc** | **NNGCAACAKAWN** |
|  | **PIF3** | **780** | **+** | **gaataaCACGTacagtta** | **GKRGGMCACGTGRMSWCK** |
|  | **GATABOX** | **635** | **+** | **GATA** | **GATA** |
|  | **GATABOX** | **658** | **-** | **TATC** | **TATC** |
|  | **GATABOX** | **53** | **-** | **TATC** | **TATC** |
|  | **GATABOX** | **418** | **+** | **GATA** | **GATA** |
|  |  |  |  |  |  |
| **MdCBF5** | **ICEr2** | **414** | **+** | **TGAGGC** | **TGAGGC** |
|  | **LTRECOREATCOR15** | **418** | **+** | **CCGAC** | **CCGAC** |
|  | **LTRE1HVBLT49** | **825** | **+** | **CCGAAA** | **CCGAAA** |
|  | **MYCCONSENSUSAT** | **570** | **+** | **CAGCTG** | **CANNTG** |
|  | **MYCCONSENSUSAT** | **685** | **+** | **CATTTG** | **CANNTG** |
|  | **MYCCONSENSUSAT** | **853** | **+** | **CACTTG** | **CANNTG** |
|  | **MYCCONSENSUSAT** | **308** | **+** | **CAGGTG** | **CANNTG** |
|  | **MYCCONSENSUSAT** | **389** | **+** | **CATGTG** | **CANNTG** |
|  | **MYCCONSENSUSAT** | **492** | **+** | **CACTTG** | **CANNTG** |
|  | **GATABOX** | **555** | **+** | **GATA** | **GATA** |
|  | **GATABOX** | **594** | **-** | **TATC** | **TATC** |
|  | **GATABOX** | **729** | **-** | **TATC** | **TATC** |
|  | **GATABOX** | **166** | **-** | **TATC** | **TATC** |
|  | **GATABOX** | **195** | **+** | **GATA** | **GATA** |
|  | **GATABOX** | **216** | **+** | **GATA** | **GATA** |
|  | **GATABOX** | **246** | **+** | **GATA** | **GATA** |
|  | **CCA1** | **778** | **+** | **AACAATCT** | **AAMAATCT** |
|  | **CIACADIANLELHC** | **780** | **+** | **CAATCTGATC** | **CAANNNNATC** |
|  | **CIACADIANLELHC** | **271** | **+** | **CAAGTTGATC** | **CAANNNNATC** |
|  | **RAV1** | **435** | **+** | **aagCAACAagat** | **NNGCAACAKAWN** |
|  | **RAV1** | **952** | **+** | **cttCAACAtcgt** | **NNGCAACAKAWN** |
|  |  |  |  |  |  |
| **MdDAM1** |  |  |  |  |  |
|  | **CBF1** | **38** | **+** | **TGGCCGAC** | **TGGCCGAC** |
|  | **LTREATLTI78** | **333** | **+** | **ACCGACA** | **ACCGACA** |
|  | **CBFHV** | **245** | **+** | **ATCGAC** | **RYCGAC** |
|  | **ACGTABREMOTIFA2OSEM** | **226** | **+** | **ACGTGTC** | **ACGTGKC** |
|  | **ACGTABREMOTIFA2OSEM** | **309** | **-** | **GACACGT** | **ACGTGKC** |
|  | **MYB1AT** | **941** | **-** | **TGGTTT** | **WAACCA** |
|  | **MYB1** | **346** | **-** | **GGTAGGAT** | **WAACCA** |
|  | **MYB2AT** | **149** | **-** | **CAGTTA** | **TAACTG** |
|  | **MYB2AT** | **78** | **+** | **TAACTG** | **TAACTG** |
|  | **PIF3** | **217** | **-** | **acctgttACGTGtcaact** | **GKRGGMCACGTGRMSWCK** |
|  | **PIF3** | **303** | **+** | **gtgggaCACGTataaag** | **NNGCAACAKAWN** |
|  | **CIACADIANLELHC** | **140** | **+** | **CAAACCGATC** | **CAANNNNATC** |
|  | **CIACADIANLELHC** | **23** | **+** | **CAAATGCATC** | **CAANNNNATC** |
|  | **CIACADIANLELHC** | **368** | **+** | **CAACTGCATC** | **CAANNNNATC** |
|  | **EVENINGAT** | **434** | **+** | **AAAATATCT** | **AAMAATCT** |
|  | **GATABOX** | **166** | **+** | **GATA** | **GATA** |
|  | **GATABOX** | **351** | **+** | **GATA** | **GATA** |
|  | **GATABOX** | **429** | **-** | **TATC** | **TATC** |
|  | **GATABOX** | **438** | **-** | **TATC** | **TATC** |
|  | **GATABOX** | **469** | **+** | **GATA** | **GATA** |
|  | **GATABOX** | **482** | **+** | **GATA** | **GATA** |
|  | **GATABOX** | **60** | **+** | **GATA** | **GATA** |
|  | **GATABOX** | **703** | **-** | **TATC** | **TATC** |
|  | **GATABOX** | **865** | **-** | **TATC** | **TATC** |
|  | **RAV1** | **415** | **+** | **gccCAACAaaat** | **NNGCAACAKAWN** |
|  | **Agamous** | **498** | **-** | **TCCATAAATGG** | **CCWWWWNNRGH** |
|  | **Agamous** | **734** | **-** | **TCTATTAATGG** | **CCWWWWNNRGH** |
|  |  |  |  |  |  |
| **MdDAM2** | **LTRECOREATCOR15** | **666** | **+** | **CCGAC** | **A/GCCGAC** |
|  | **DRE1COREZMRAB17** | **304** | **+** | **ACCGAGA** | **ACCGAGA** |
|  | **ABRERATCAL** | **429** | **-** | **CCACGTT** | **MACGYGB** |
|  | **MYB1AT** | **613** | **-** | **TGGTTT** | **WAACCA** |
|  | **MYB1AT** | **716** | **-** | **TGGTTT** | **WAACCA** |
|  | **MYB1AT** | **949** | **-** | **TGGTTT** | **WAACCA** |
|  | **MYB1AT** | **8** | **+** | **AAACCA** | **WAACCA** |
|  | **MYB2AT** | **922** | **-** | **CAGTTA** | **TAACTG** |
|  | **MYB2AT** | **315** | **-** | **CAGTTA** | **TAACTG** |
|  | **PIF3** | **416** | **+** | **gcgttcCACGTttcttac** | **GKRGGMCACGTGRMSWCK** |
|  | **EVENINGAT** | **446** | **+** | **AAAATATCT** | **AAMAATCT** |
|  | **GATABOX** | **865** | **-** | **TATC** | **TATC** |
|  | **GATABOX** | **246** | **+** | **GATA** | **GATA** |
|  | **GATABOX** | **321** | **+** | **GATA** | **GATA** |
|  | **GATABOX** | **450** | **-** | **TATC** | **TATC** |
|  | **GATABOX** | **482** | **+** | **GATA** | **GATA** |
|  | **GATABOX** | **495** | **+** | **GATA** | **GATA** |
|  | **RAV1** | **202** | **-** | **caagTGTTGcag** | **NNGCAACAKAWN** |
|  | **RAV1** | **769** | **-** | **ggctTGTTGggc** | **NNGCAACAKAWN** |
|  |  |  |  |  |  |
| **MdDAM3** | **ABRE-like** | **219** | **+** | **GACGTGGA** | **BACGTGKM** |
|  | **MYB1AT** | **411** | **+** | **AAACCA** | **WAACCA** |
|  | **MYB1AT** | **830** | **-** | **TGGTTA** | **WAACCA** |
|  | **MYB2AT** | **984** | **+** | **TAACTG** | **TAACTG** |
|  | **PIF3** | **213** | **-** | **ggtaaagACGTGgaaacg** | **GKRGGMCACGTGRMSWCK** |
|  | **CCA1** | **726** | **-** | **AGATTTTT** | **AAMAATCT** |
|  | **CIACADIANLELHC** | **491** | **+** | **CAAAACAATC** | **CAANNNNATC** |
|  | **GATABOX** | **138** | **+** | **GATA** | **GATA** |
|  | **GATABOX** | **157** | **-** | **TATC** | **TATC** |
|  | **GATABOX** | **21** | **-** | **TATC** | **TATC** |
|  | **GATABOX** | **385** | **-** | **TATC** | **TATC** |
|  | **GATABOX** | **520** | **-** | **TATC** | **TATC** |
|  | **GATABOX** | **668** | **-** | **TATC** | **TATC** |
|  | **GATABOX** | **929** | **-** | **TATC** | **TATC** |
|  | **RAV1** | **130** | **-** | **agagTGTTGata** | **NNGCAACAKAWN** |
|  | **RAV1** | **176** | **+** | **gctCAACAtttg** | **NNGCAACAKAWN** |
|  | **RAV1** | **195** | **+** | **cgcCAACAgcaa** | **NNGCAACAKAWN** |
|  | **RAV1** | **462** | **+** | **cgtCAACAaatc** | **NNGCAACAKAWN** |
|  | **RAV1** | **485** | **+** | **aatCAACAaaac** | **NNGCAACAKAWN** |
|  | **RAV1** | **568** | **-** | **gactTGTTGgtc** | **NNGCAACAKAWN** |
|  | **Agamous** | **253** | **+** | **CCATAAAAAGC** | **CCWWWWNNRGH** |
|  | **Agamous** | **396** | **+** | **CCAAATACAGC** | **CCWWWWNNRGH** |
|  | **Agamous** | **533** | **-** | **TCTCCAATTGG** | **CCWWWWNNRGH** |
|  | **Agamous** | **735** | **-** | **GCTAAATTTGG** | **CCWWWWNNRGH** |
|  | **Agamous** | **823** | **+** | **CCTTTTCTGGT** | **CCWWWWNNRGH** |
|  | **Agamous** | **901** | **+** | **CCAAAACAAGC** | **CCWWWWNNRGH** |
|  |  |  |  |  |  |
| **MdRGL1a** | **LTRECOREATCOR15** | **281** | **+** | **CCGAC** | **A/GCCGAC** |
| **DELLA** | **LTRECOREATCOR15** | **435** | **+** | **CCGAC** | **A/GCCGAC** |
|  | **MYB1AT** | **855** | **+** | **AAACCA** | **WAACCA** |
|  | **MYB1AT** | **118** | **+** | **AAACCA** | **WAACCA** |
|  | **MYB2AT** | **785** | **+** | **TAACTG** | **TAACTG** |
|  | **PIF3** | **296** | **+** | **aagtctCACGTttgagat** | **GKRGGMCACGTGRMSWCK** |
|  | **CIACADIANLELHC** | **515** | **+** | **CAATCAAATC** | **CAANNNNATC** |
|  | **GATABOX** | **636** | **-** | **TATC** | **TATC** |
|  | **GATABOX** | **709** | **+** | **GATA** | **GATA** |
|  | **GATABOX** | **957** | **-** | **TATC** | **TATC** |
|  | **GATABOX** | **13** | **-** | **TATC** | **TATC** |
|  | **GATABOX** | **54** | **-** | **TATC** | **TATC** |
|  | **GATABOX** | **89** | **-** | **TATC** | **TATC** |
|  | **GATABOX** | **227** | **-** | **TATC** | **TATC** |
|  | **GATABOX** | **311** | **+** | **GATA** | **GATA** |
|  | **GATABOX** | **417** | **+** | **GATA** | **GATA** |
|  | **RAV1** | **124** | **+** | **accCAACAaaaa** | **NNGCAACAKAWN** |
|  | **RAV1** | **242** | **+** | **cgcCAACAtata** | **NNGCAACAKAWN** |
|  | **RAV1** | **968** | **+** | **attCAACAaaac** | **NNGCAACAKAWN** |
|  |  |  |  |  |  |
| **MdRGL1b** | **ABRERATCAL** | **354** | **+** | **CACGTGC** | **MACGYGB** |
| **DELLA** | **MYB1AT** | **852** | **+** | **AAACCA** | **WAACCA** |
|  | **MYB1AT** | **202** | **-** | **TGGTTT** | **WAACCA** |
|  | **MYB2AT** | **812** | **+** | **TAACTG** | **TAACTG** |
|  | **PIF3** | **327** | **+** | **ccaaatCACGTgcctgta** | **GKRGGMCACGTGRMSWCK** |
|  | **GATABOX** | **511** | **+** | **GATA** | **GATA** |
|  | **GATABOX** | **597** | **-** | **TATC** | **TATC** |
|  | **GATABOX** | **660** | **-** | **TATC** | **TATC** |
|  | **GATABOX** | **74** | **-** | **TATC** | **TATC** |
|  | **GATABOX** | **119** | **+** | **GATA** | **GATA** |
|  | **GATABOX** | **209** | **-** | **TATC** | **TATC** |
|  | **GATABOX** | **300** | **-** | **TATC** | **TATC** |
|  | **RAV1** | **215** | **-** | **gttcTGTTGaaa** | **NNGCAACAKAWN** |
|  | **RAV1** | **615** | **-** | **ataaTGTTGttt** | **NNGCAACAKAWN** |
|  | **RAV1** | **647** | **-** | **aggtTGTTGttt** | **NNGCAACAKAWN** |
|  | **RAV1** | **737** | **+** | **tttCAACAttca** | **NNGCAACAKAWN** |
|  | **RAV1** | **925** | **-** | **aattTGTTGcaa** | **NNGCAACAKAWN** |
|  | **RAV1** | **942** | **+** | **attCAACAaaat** | **NNGCAACAKAWN** |
|  |  |  |  |  |  |
| **MdRGL2a** | **LTRECOREATCOR15** | **455** | **-** | **GTCGG** | **A/GCCGAC** |
| **DELLA** | **LTRE1HVBLT49** | **244** | **-** | **CCGAAA** | **CCGAAA** |
|  | **MYB1AT** | **278** | **+** | **AAACCA** | **WAACCA** |
|  | **MYB2CONSENSUSAT** | **337** | **+** | **CAACTG** | **YAACKG** |
|  | **GATABOX** | **778** | **-** | **TATC** | **TATC** |
|  | **GATABOX** | **955** | **-** | **TATC** | **TATC** |
|  | **GATABOX** | **52** | **+** | **GATA** | **GATA** |
|  | **GATABOX** | **212** | **-** | **TATC** | **TATC** |
|  | **GATABOX** | **343** | **+** | **GATA** | **GATA** |
|  | **RAV1** | **251** | **+** | **actCAACAgttt** | **NNGCAACAKAWN** |
|  | **RAV1** | **498** | **+** | **ataCAACAcata** | **NNGCAACAKAWN** |
|  | **RAV1** | **903** | **+** | **gacCAACAtgca** | **NNGCAACAKAWN** |
|  |  |  |  |  |  |
| **MdRGL2b** | **ABREATRD22** | **707** | **-** | **CACCACGTAT** | **RYACGTGGYR** |
| **DELLA** | **ABRERATCAL** | **794** | **-** | **ACACGTG** | **MACGYGB** |
|  | **ABRERATCAL** | **10** | **+** | **AACGTGG** | **MACGYGB** |
|  | **MYB1AT** | **154** | **+** | **AAACCA** | **WAACCA** |
|  | **MYB2AT** | **106** | **-** | **CAGTTA** | **TAACTG** |
|  | **PIF3** | **4** | **-** | **ttttgtaACGTGgcggtg** | **GKRGGMCACGTGRMSWCK** |
|  | **PIF3** | **510** | **+** | **nnnnnaCACGTaatgtac** | **GKRGGMCACGTGRMSWCK** |
|  | **PIF3** | **701** | **+** | **gtacacCACGTatcgcta** | **GKRGGMCACGTGRMSWCK** |
|  | **PIF3** | **786** | **+** | **tacaaaCACGTgatgtac** | **GKRGGMCACGTGRMSWCK** |
|  | **PIF3** | **786** | **-** | **tacaaacACGTGatgtac** | **GKRGGMCACGTGRMSWCK** |
|  | **GATABOX** | **559** | **+** | **GATA** | **GATA** |
|  | **GATABOX** | **590** | **+** | **GATA** | **GATA** |
|  | **GATABOX** | **643** | **+** | **GATA** | **GATA** |
|  | **GATABOX** | **714** | **-** | **TATC** | **TATC** |
|  | **GATABOX** | **734** | **+** | **GATA** | **GATA** |
|  | **GATABOX** | **957** | **-** | **TATC** | **TATC** |
|  | **GATABOX** | **99** | **+** | **GATA** | **GATA** |
|  | **GATABOX** | **234** | **-** | **TATC** | **TATC** |
|  | **RAV1** | **806** | **-** | **cttgTGTTGggt** | **NNGCAACAKAWN** |
|  | **RAV1** | **888** | **+** | **ctgCAACAatta** | **NNGCAACAKAWN** |
|  | **RAV1** | **919** | **+** | **gacCAACAtgga** | **NNGCAACAKAWN** |
|  |  |  |  |  |  |
| **MdRGL3a** | **CIACADIANLELHC** | **442** | **+** | **CAACATTATC** | **CAANNNNATC** |
| **DELLA** | **GATABOX** | **711** | **-** | **TATC** | **TATC** |
|  | **GATABOX** | **940** | **-** | **TATC** | **TATC** |
|  | **GATABOX** | **956** | **-** | **TATC** | **TATC** |
|  | **GATABOX** | **125** | **-** | **TATC** | **TATC** |
|  | **GATABOX** | **162** | **-** | **TATC** | **TATC** |
|  | **GATABOX** | **331** | **+** | **GATA** | **GATA** |
|  | **GATABOX** | **368** | **+** | **GATA** | **GATA** |
|  | **GATABOX** | **448** | **-** | **TATC** | **TATC** |
|  | **RAV1** | **224** | **-** | **aggtTGTTGttt** | **NNGCAACAKAWN** |
|  | **RAV1** | **428** | **+** | **tgtCAACAttat** | **NNGCAACAKAWN** |
|  |  |  |  |  |  |
| **MdRGL3b** | **ABREATRD22** | **933** | **-** | **CACCACGTAT** | **RYACGTGGYR** |
| **DELLA** | **MYB2CONSENSUSAT** | **751** | **-** | **CAGTTG** | **YAACKG** |
|  | **MYB2CONSENSUSAT** | **784** | **-** | **CAGTTG** | **YAACKG** |
|  | **MYBATRD22** | **457** | **+** | **CTAACCA** | **CTAACCA** |
|  | **PIF3** | **278** | **-** | **ttatagtACGTGaattta** | **GKRGGMCACGTGRMSWCK** |
|  | **PIF3** | **640** | **+** | **tataaaCACGTattgatc** | **GKRGGMCACGTGRMSWCK** |
|  | **PIF3** | **926** | **+** | **tcccacCACGTatctgaa** | **GKRGGMCACGTGRMSWCK** |
|  | **GATABOX** | **662** | **+** | **GATA** | **GATA** |
|  | **GATABOX** | **940** | **-** | **TATC** | **TATC** |
|  | **GATABOX** | **956** | **-** | **TATC** | **TATC** |
|  | **GATABOX** | **996** | **-** | **TATC** | **TATC** |
|  | **GATABOX** | **104** | **-** | **TATC** | **TATC** |
|  | **GATABOX** | **181** | **-** | **TATC** | **TATC** |
|  | **GATABOX** | **222** | **+** | **GATA** | **GATA** |
|  | **GATABOX** | **238** | **+** | **GATA** | **GATA** |
|  | **GATABOX** | **358** | **-** | **TATC** | **TATC** |
|  | **RAV1** | **374** | **+** | **attCAACAttca** | **NNGCAACAKAWN** |
|  | **RAV1** | **381** | **+** | **attCAACAttca** | **NNGCAACAKAWN** |
|  | **Agamous** | **134** | **+** | **CCAAAAGGAGA** | **CCWWWWNNRGH** |
|  |  |  |  |  |  |
| **MdEBB1** | **LTRECOREATCOR15** | **383** | **+** | **CCGAC** | **A/GCCGAC** |
|  | **MYB1AT** | **545** | **+** | **AAACCA** | **WAACCA** |
|  | **GATABOX** | **325** | **-** | **TATC** | **TATC** |
|  | **GATABOX** | **325** | **-** | **TATC** | **TATC** |
|  | **GATABOX** | **965** | **-** | **TATC** | **TATC** |
|  | **GATABOX** | **965** | **-** | **TATC** | **TATC** |
|  | **GATABOX** | **972** | **+** | **GATA** | **GATA** |
|  | **GATABOX** | **972** | **+** | **GATA** | **GATA** |
|  | **GATABOX** | **982** | **-** | **TATC** | **TATC** |
|  | **GATABOX** | **982** | **-** | **TATC** | **TATC** |
|  | **RAV1** | **394** | **+** | **atcCAACAactt** | **NNGCAACAKAWN** |
|  | **RAV1** | **575** | **-** | **tttgTGTTGcat** | **NNGCAACAKAWN** |
|  | **RAV1** | **632** | **+** | **ttaCAACActac** | **NNGCAACAKAWN** |
|  | **RAV1** | **938** | **+** | **catCAACAaacc** | **NNGCAACAKAWN** |

Table S2 Legend:

| Promoter elements found in *MdCBFs1-5, MdRGLs1-3,* putative *MdDAMs* and putative *MdEBB* genes. The 5′ 1000 bp upstream of the putative translational start site were analyzed by PLACE (<http://www.dna.affrc.go.jp/PLACE/>, 15 October, 2014 date last accessed; Higo et al. 1999), PAN ([http://plantpan.mbc.nctu.edu.tw/gene_group/index. php](http://plantpan.mbc.nctu.edu.tw/gene_group/index.%20php), 15 October, 2014 date last accessed; Chang et al. 2008) and PLANTCARE (<http://bioinformatics.psb.ugent.be/webtools/plantcare/html/>, 15 October, 2014 date last accessed; Lescot et al. 2002). Position is starting 1000 bp from translational start site; (+) or (−) indicates strand. Sequence in the promoter is as indicated, while published consensus sequence data are from the PLACE, PAN and PLANTCARE databases. The promoter elements for each gene are arranged in the following order: low-temperature response (LTRE), *CBF*-specific regulatory motifs (MYC, CM, CAMTA, ICEr1, ICEr2), abscisic acid or dehydration response elements (MYB or ABRE) and light-responsive elements (evening element, GATA, CCA and PIF), floral or bud development (RAV1 or AGAMOUS). Standard genetic code; R = A/G, Y = C/T, M = A/C, K = G/T, S = C/G, W = A/T, B = C/G/T, V = A/C/G. |
| --- |
