# Supplementary figures and images for: Overexpression of a peach CBF gene in apple: a model for understanding the integration of growth, dormancy, and cold hardiness in woody plants
Source: Front Plant Sci. 2015 Feb 27;6:85. doi: 10.3389/fpls.2015.00085 (PMC4343015; doi:10.3389/fpls.2015.00085)

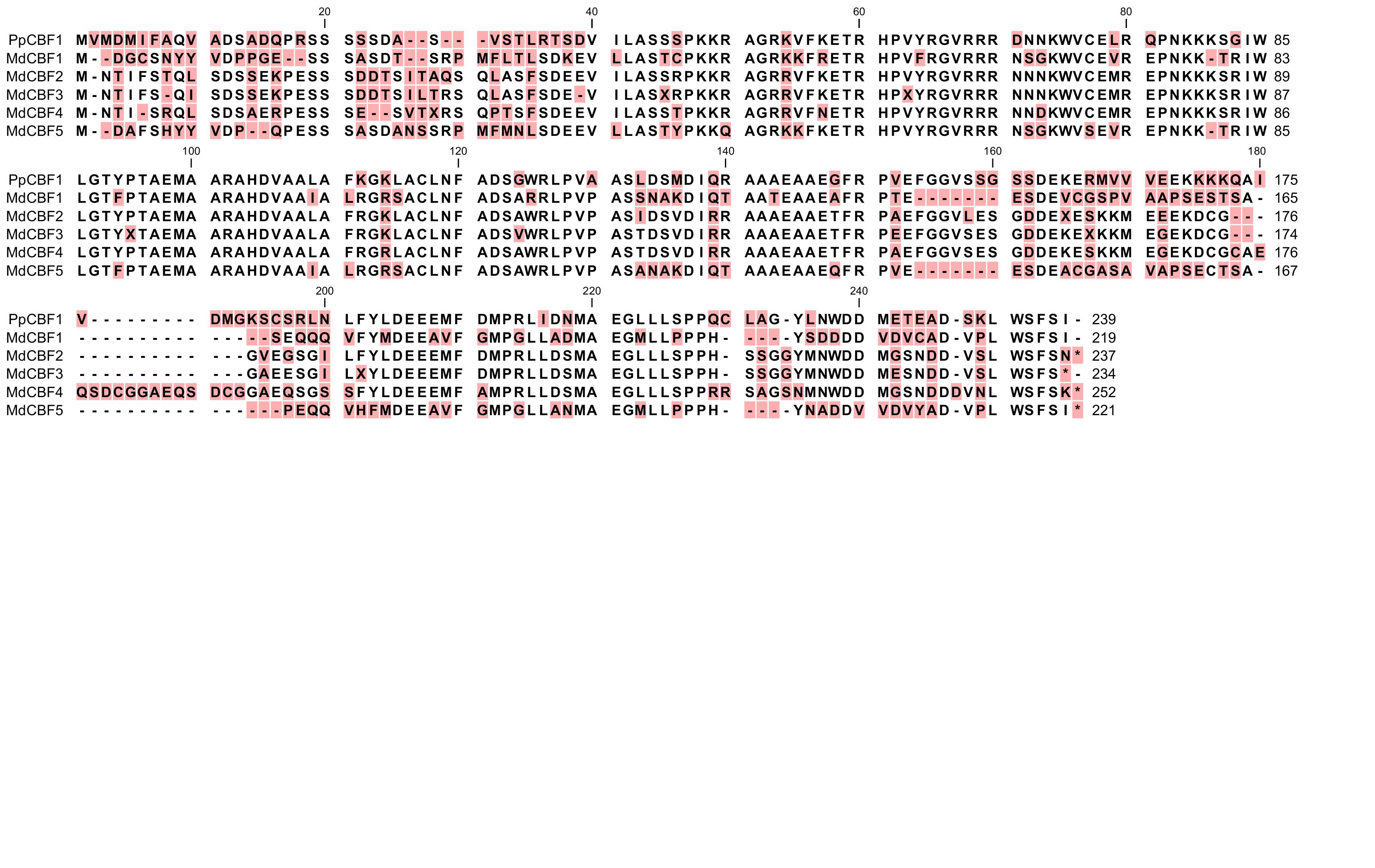

Supplement: Figure S1 — Alignment of conceptual MdCBF1-5 and PpCBF1 amino acid sequences. Light red residues indicate different residues between the sequences. The alignment was performed with CLUSTALW (Thompson et al., 1994). [file DataSheet1.ZIP › Image 1.JPEG]

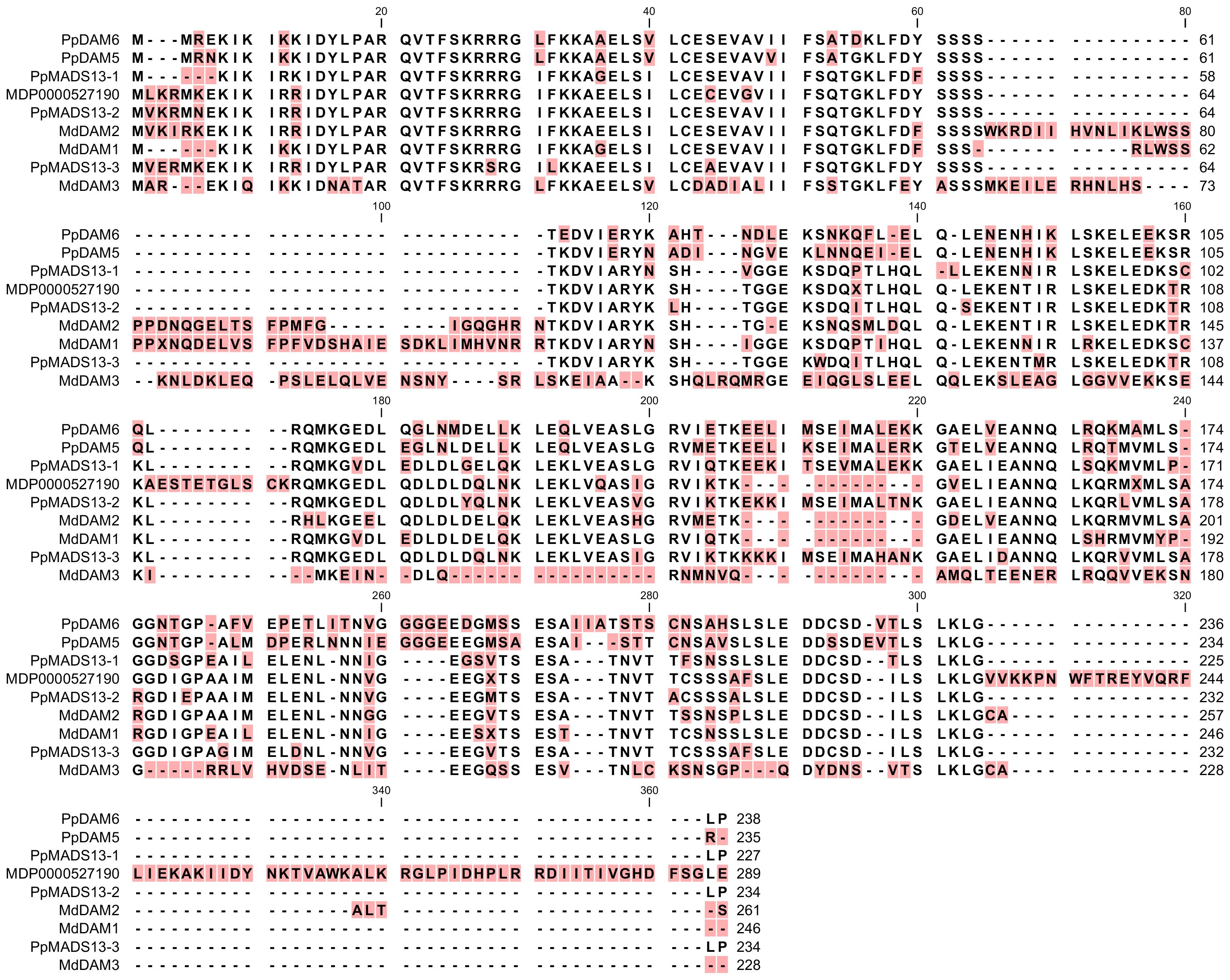

Supplement: Figure S1 — Alignment of conceptual MdCBF1-5 and PpCBF1 amino acid sequences. Light red residues indicate different residues between the sequences. The alignment was performed with CLUSTALW (Thompson et al., 1994). [file DataSheet1.ZIP › Image 2.JPEG]

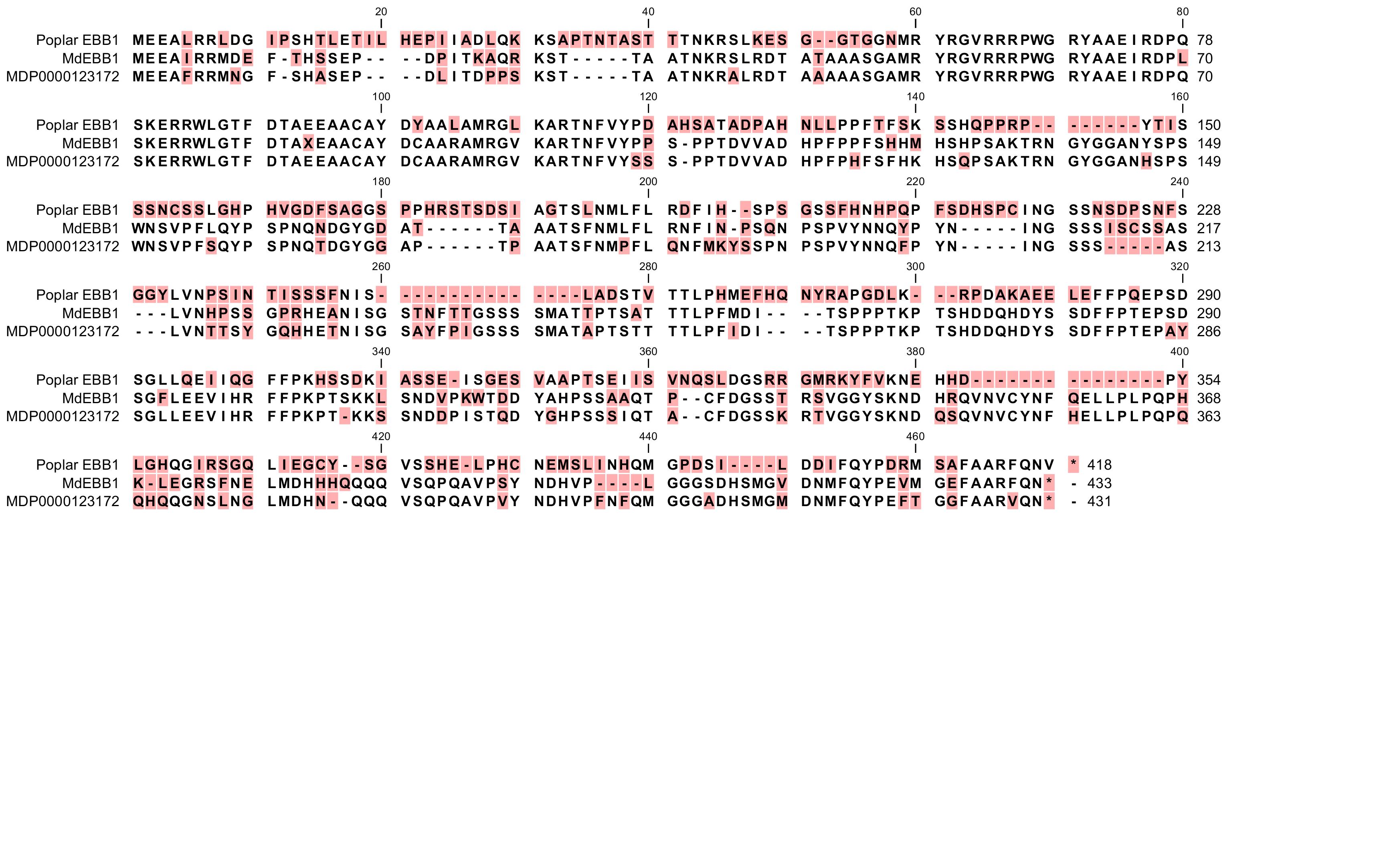

Supplement: Figure S1 — Alignment of conceptual MdCBF1-5 and PpCBF1 amino acid sequences. Light red residues indicate different residues between the sequences. The alignment was performed with CLUSTALW (Thompson et al., 1994). [file DataSheet1.ZIP › Image 3.JPEG]

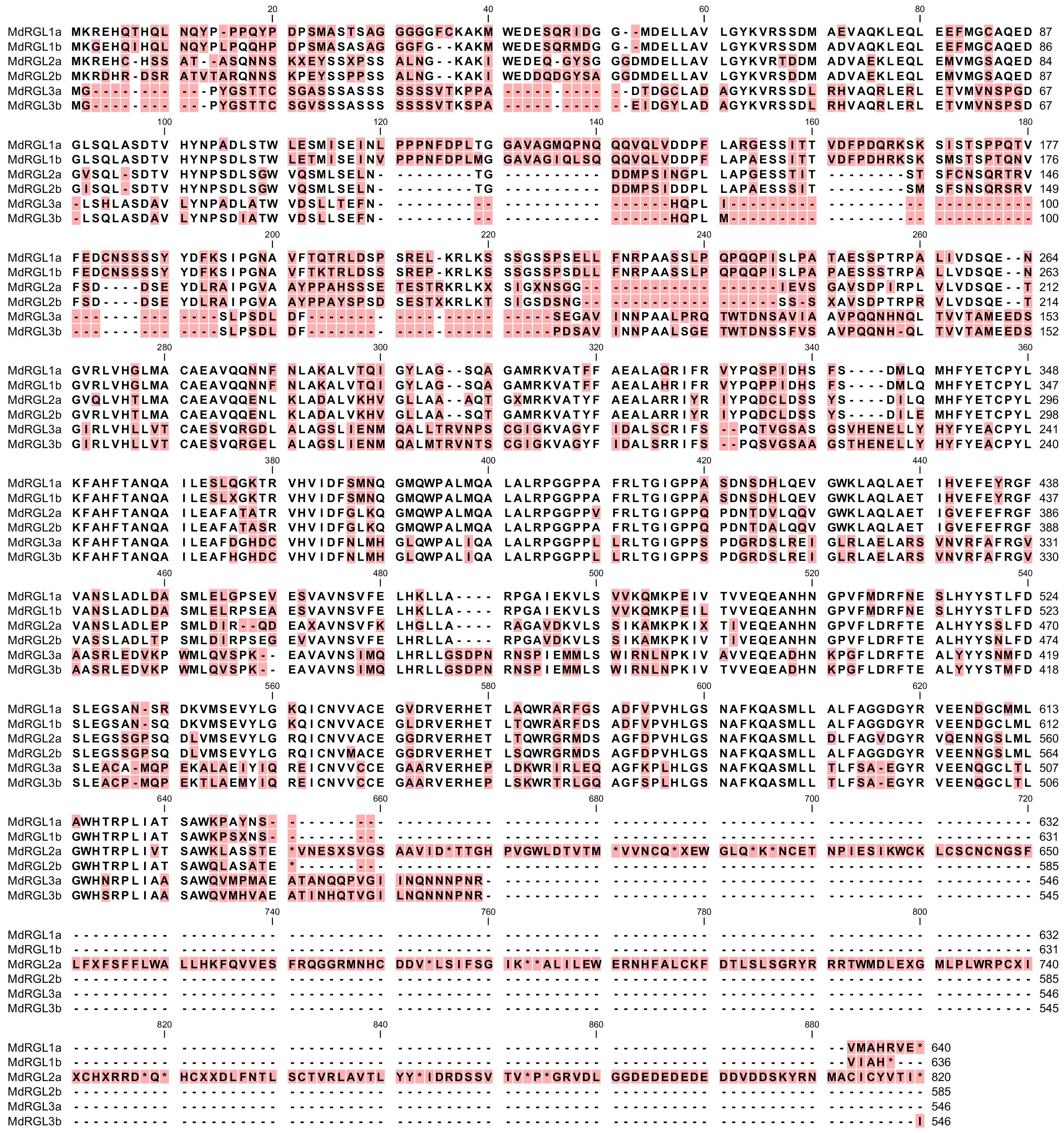

Supplement: Figure S1 — Alignment of conceptual MdCBF1-5 and PpCBF1 amino acid sequences. Light red residues indicate different residues between the sequences. The alignment was performed with CLUSTALW (Thompson et al., 1994). [file DataSheet1.ZIP › Image 4.JPEG]
